# Distinct thalamocortical network dynamics are associated with the pathophysiology of chronic low back pain

**DOI:** 10.1038/s41467-020-17788-z

**Published:** 2020-08-07

**Authors:** Yiheng Tu, Zening Fu, Cuiping Mao, Maryam Falahpour, Randy L. Gollub, Joel Park, Georgia Wilson, Vitaly Napadow, Jessica Gerber, Suk-Tak Chan, Robert R. Edwards, Ted J. Kaptchuk, Thomas Liu, Vince Calhoun, Bruce Rosen, Jian Kong

**Affiliations:** 1 Department of Psychiatry, Massachusetts General Hospital, Harvard Medical School, Charlestown, MA USA; 2 Department of Radiology, Martinos Center for Biomedical Imaging, Massachusetts General Hospital, Harvard Medical School, Charlestown, MA USA; 3 Tri-Institutional Center for Translational Research in Neuroimaging and Data Science (TReNDS), Georgia State University, Georgia Institute of Technology, Emory University, Atlanta, GA USA; 4 Department of Medical Imaging, First Affiliated Hospital of Xi’An Jiao Tong University College of Medicine, Xi’an, Shaan’Xi China; 5 Center for Functional MRI, University of California San Diego, La Jolla, CA USA; 6 Department of Anesthesiology, Perioperative and Pain Medicine, Brigham and Women’s Hospital, Harvard Medical School, Boston, MA USA; 7 Beth Israel Deaconess Medical Center, Harvard Medical School, Boston, MA USA

**Keywords:** Chronic pain, Chronic pain

## Abstract

Thalamocortical dysrhythmia is a key pathology of chronic neuropathic pain, but few studies have investigated thalamocortical networks in chronic low back pain (cLBP) given its non-specific etiology and complexity. Using fMRI, we propose an analytical pipeline to identify abnormal thalamocortical network dynamics in cLBP patients and validate the findings in two independent cohorts. We first identify two reoccurring dynamic connectivity states and their associations with chronic and temporary pain. Further analyses show that cLBP patients have abnormal connectivity between the ventral lateral/posterolateral nucleus (VL/VPL) and postcentral gyrus (PoCG) and between the dorsal/ventral medial nucleus and insula in the less frequent connectivity state, and temporary pain exacerbation alters connectivity between the VL/VPL and PoCG and the default mode network in the more frequent connectivity state. These results extend current findings on thalamocortical dysfunction and dysrhythmia in chronic pain and demonstrate that cLBP pathophysiology and clinical pain intensity are associated with distinct thalamocortical network dynamics.

## Abstract (web summary)

Thalamocortical dysrhythmia is a key pathology of chronic pain. Here, the authors propose an analytical pipeline to study dynamic fMRI brain networks and demonstrate that chronic low back pain pathophysiology and clinical pain intensity are associated with distinct thalamocortical network dynamics.

## Introduction

Chronic low back pain (cLBP) is the number one cause of disability globally^1^, and the problem is worsening due to an aging and increasing world population^2^. Current treatment regimens are ineffective in a significant number of individuals, and few nonopioid and nonaddictive pain medications have been developed over the past five decades^3^. cLBP is characterized by a range of biophysical, psychological, and social factors with extreme variability in genesis^4^. This complexity and the limited understanding of the neural mechanisms responsible for the development, maintenance, and experience of cLBP hinder the development of new treatments.

The brain of the chronic pain patient is continuously processing background pain by integrating information between multiple brain regions related to sensory, cognitive, and emotional functions^5^. Multiple lines of evidence suggest a critical role of the thalamus in chronic pain processing. Electrophysiological studies have shown altered thalamocortical rhythm, termed thalamocortical dysrhythmia (TCD)^6^, in chronic pain^7–9^. The abnormal, internally generated low-frequency oscillations in the thalamo-cortico-thalamic network disrupts the normal state-dependent flow of information between the thalamus and cortex. This in turn leads to disturbances of sensation, motor performance, and cognition in patients with chronic pain^7^. Studies using functional magnetic resonance imaging (fMRI) have also demonstrated that abnormal low-frequency oscillations and connectivity in thalamocortical networks underpin the constant perception of pain^10,11^. However, this evidence is mainly found in neuropathic pain (NP), and the role of thalamocortical networks in cLBP is still unknown. Unlike NP, cLBP is not necessarily sustained by peripheral nerve injury, and rarely can a specific cause of cLBP be identified^4^. Investigating the neural mechanisms of thalamocortical networks in cLBP is challenging but important for potential therapeutic targets.

In past decades, fMRI resting-state functional connectivity has provided high spatial resolution for studying brain networks. Challenging the conventional assumption that functional interactions remain constant throughout the entire resting-state scan, recent studies have shown that rsFC can vary considerably in different temporal scales^12–15^, and such time-varying characteristics may represent spontaneous alterations in the underlying networks and thus may reveal neural mechanisms that cannot be discovered through static rsFC alone^16–18^.

In this study, we examined dynamic rsFC in 90 cLBP patients and 74 healthy controls (HCs) using fMRI. We hypothesized that cLBP would be associated with dynamic connectivity abnormalities of the thalamocortical networks, which would be correlated with clinical symptoms. Patients underwent two resting-state fMRI scans before and after physical maneuvers aimed to exacerbate their spontaneous LBP. This allowed us to separate brain patterns associated with chronic pain pathophysiology (as compared with HCs) and temporal intensity alternations of clinical pain (high pain vs. low pain)^19,20^ since previous studies have suggested that neural dynamics discriminating cLBP patients from HCs may be distinct from neural dynamics sensitive to pain intensity changes^20,21^. We applied a novel analytical framework combining sliding-window cross-correlation, clustering state analysis, and graph-theory methods to capture abnormal thalamocortical network dynamics and their relationships with clinical symptoms in cLBP patients under two different conditions (i.e., low and high spontaneous LBP). The global and local efficiency of information transfer in large scale brain networks was also investigated and compared between cLBP and HCs. In addition, we tested the validity of the findings using an independent dataset consisting of 30 cLBP patients (each patient had two fMRI scans separated by about 2 weeks to explore test–retest reliability) and 30 HCs. We also replicated the findings from a dataset of 25 cLBP patients and 25 HCs with a similar attention/vigilance level, as demonstrated by a multisource interference task (MSIT), to rule out the confounding effect of vigilance.

## Results

### Demographics and clinical scores for cLBP patients

For exploration and validation, we used three independent cohorts of subjects (Table 1). The first cohort (Dataset 1) consisted of 90 cLBP patients (age 34.5 ± 9.0; 38 males) and 74 HCs (age 32.4 ± 8.4; 31 males). Patients had an average pain severity (using the Pain Bothersomeness visual analog scale [VAS] from 0, “not at all bothersome,” to 10, “extremely bothersome”) of 5.1 ± 1.9 during the past 7 days. Pain severity was the primary clinical measure in this study, as previous studies^21,22^ and our research^23^ have suggested that cLBP may modulate brain functions beyond the pain system itself in ways that may be maladaptive, affecting patients’ daily experiences and diminishing their quality of life. In addition, patients were required to rate their current pain intensity (numerical rating scale [NRS] from 0, “no pain,” to 100, “worst pain imaginable”) prior to the two resting-state fMRI scans, between which they performed pain-exacerbating maneuvers to increase their LBP intensity. Across 90 cLBP patients, the current pain intensities were increased from 31.7 ± 20.1 to 51.5 ± 20.1, while 14 of them had the same or decreased levels of pain intensity. These 14 patients were excluded from the following fMRI analyses comparing dynamic rsFC and thalamocortical networks between low-pain and exacerbated-pain conditions. The remaining 76 patients had current pain intensities increased from 31.3 ± 18.7 to 55.2 ± 19.6.

**Table 1:** Demographics (mean ± SD) of cLBP and HCs.

Characteristics	Dataset 1		Dataset 2		Dataset 3	
	cLBP (*n* = 90)	HCs (*n* = 74)	cLBP (*n* = 30)	HCs (*n* = 30)	cLBP (*n* = 25)	HCs (*n* = 25)
Age (years)	34.5 ± 9.0	32.4 ± 8.4	37.2 ± 11.0	33.5 ± 7.2	48.0 ± 9.6	44.3 ± 12.2
Gender (male/female)	38/52	31/43	13/17	17/13	7/18	9/16
Pain duration (years)	6.9 ± 6.2	NA	5.9 ± 7.1	NA	6.1 ± 5.8	NA

The second cohort (Dataset 2) consisted of 30 cLBP patients (age 37.2 ± 11.0; 13 males) and 30 HCs (age 33.5 ± 7.2; 17 males). Patients had an average pain intensity of 5.8 ± 1.5 (0–10 NRS) for the past 7 days before the first resting-state fMRI scan, and an average current pain intensity of 44.0 ± 23.1 and 44.1 ± 23.1 (0–100 NRS) prior to the first and second resting-state fMRI scans, respectively (these two sessions were separated by about 2 weeks). Please note that one of the independent cohorts measured pain severity using the Pain Bothersomeness scale over the past 7 days (Dataset 1) and the other cohort measured pain intensity using the NRS over the past 7 days (Dataset 2). Although each cohort used a different measure, pain intensity and pain severity are likely to be highly correlated and both were used to distinguish chronic pain from acute clinical pain (i.e., current pain intensity).

The third cohort (Dataset 3) consisted of 25 cLBP patients (age 48.0 ± 9.6; 7 males) and 25 HCs (age 44.3 ± 12.2; 9 males) from an independent site that performed a MSIT^24^ (see “Cross-site validation” for details). Patients had an average past pain intensity of 6.1 ± 5.8 (0–10 NRS).

### Brain parcellation and whole-brain connectivity estimation

**Fig. 1:**
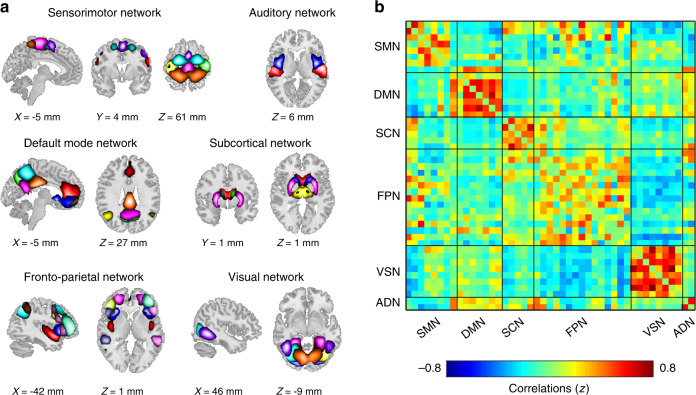
Spatial maps of the identified intrinsic ICNs and static connectivity matrix. **a** 45 ICNs were identified and sorted into six resting-state networks. Each color represents a single ICN. **b** Group-averaged static functional connectivity matrix. ICNs intrinsic connectivity networks, SMN sensorimotor network, DMN default mode network, SCN subcortical network, FPN frontoparietal network, VSN visual network, ADN auditory network.

We parcellated the brain into regions and networks of interest using group independent component analysis (GICA), a powerful data-driven approach for capturing individual differences of real functional boundaries in the brain^25^. Previous studies have typically conducted GICA on the exploratory dataset and have identified targeted independent components (ICs) as intrinsic connectivity networks (ICNs). However, due to the differences between datasets (e.g., sample size, data dimensions, or data qualities), the traditional GICA-identified ICNs are variable and not necessarily consistent across studies, which limits the replicability of the findings. In this study, we adopted a method capable of identifying reliable ICNs that can be compared across datasets^26^. The group template of ICNs was identified from two independent datasets (Human Connectome Project [HCP] and Genomics Superstruct Project [GSP]) with large sample sizes (*N* = 1005 for GSP and *N* = 823 for HCP) and different temporal resolutions (see details in Supplementary Note 1), and was used as a reference within a spatially constrained ICA algorithm^27,28^ to compute individual spatial maps and time courses for Dataset 1, Dataset 2, and Dataset 3. The 45 replicable ICNs were categorized into six functional networks (Fig. 1a), including the sensorimotor network (SMN)^20,29^, default mode network (DMN)^30,31^, frontoparietal network (FPN)^32^, subcortical network (SCN)^33^, visual network (VSN)^34^, and auditory network^22^, which have been widely studied in chronic pain. The detailed component labels and peak coordinates of each ICN are provided in Supplementary Figs. 1–6 and Supplementary Table 1.

Four post-processing steps were performed on time courses of ICNs to remove remaining noise sources, including (1) detrending linear, quadratic, and cubic trends; (2) nuisance regression of head-motion-related points detected by artifact detection toolbox (ART) in fMRI preprocessing; (3) de-spiking detected outliers; and (4) low-pass filtering with a cutoff frequency of 0.15 Hz. After these additional quality control steps, we calculated 45 × 45 correlation matrices for each subject and applied the Fisher *z*-transformation to each correlation coefficient, resulting in static functional network connectivity (sFNC). We did not observe strong thalamocortical connectivity across participants (Fig. 1b).

To further increase the validity of our study, we tested an additional parcellation strategy of using the established and extensively validated functional atlas by Yeo and colleagues^35^. In addition to the Yeo atlas, we added six regions in the SCN (bilateral thalamus, bilateral caudate, and bilateral putamen) and six regions in the SMN (bilateral postcentral gyrus [PoCG], bilateral precentral gyrus, and bilateral paracentral lobule [ParaCL]) from the automated anatomical labeling (AAL) atlas to match the regions identified from GICA. Details of the parcellation can be found in Supplementary Note 2 and Supplementary Fig. 7.

### Clustering analysis and dynamic functional connectivity

**Fig. 2:**
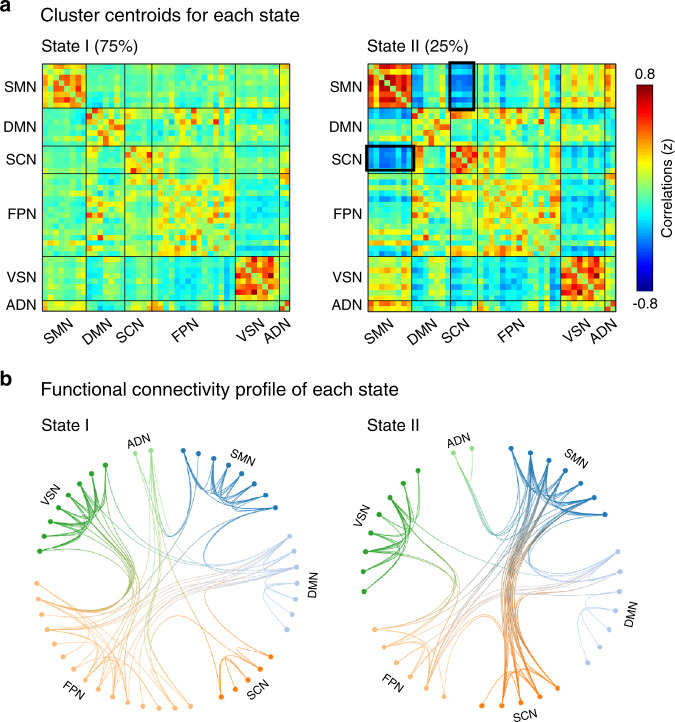
Findings of clustering analysis. **a** Cluster centroids of each state. Two states, a more frequent (around 75% of total occurrences) and sparsely connected State 1, and a less frequent (around 25% of total occurrences) and more strongly interconnected State 2. **b** Top 100 of the functional connectivity in each state, representing the strongest connections (i.e., the absolute value of correlation coefficients). Each color represents one of the six networks. Between-network connections were indicated by a transition of colors between the two networks.

We calculated dynamic functional network connectivity (dFNC) among ICNs using a sliding-window approach with graphical LASSO and then conducted a *k*-mean clustering on the dFNC estimates to identify recurring functional states^14,36^. We performed a cluster number validity analysis using a silhouette method to identify the optimal number of clusters ranging from 2 to 10^37^, and it was determined to be 2 (Supplementary Fig. 8). Two highly structured dFNC states that reoccurred throughout individual scans and across participants were identified (Fig. 2a): a more frequent (around 75% of total occurrences) and sparsely connected State 1, and a less frequent (around 25% of total occurrences) and more strongly interconnected State 2. Figure 2b shows the top 100 (as indexed by the absolute strength of dFNC) connections in two states. In State 1, connections between ICNs were located mainly within the SMN and VSN and between the FPN and other networks. The connectivity pattern of State 1 was very similar to the pattern of sFNC in Fig. 1b. In contrast, State 2 was mainly characterized by strong connectivity between the SCN (including the thalamus, putamen, and caudate) and SMN (including the PoCG, precentral gyrus, and ParaCL). Details of the connections (with labels of ICNs) in these two states can be found in Supplementary Fig. 9. We validated this finding using the Yeo atlas (Supplementary Fig. 10), indicating that the time-varying characteristics of rsFC might be consistent under different brain parcellation strategies.

### Group difference of occurrences and dFNC patterns

**Fig. 3:**
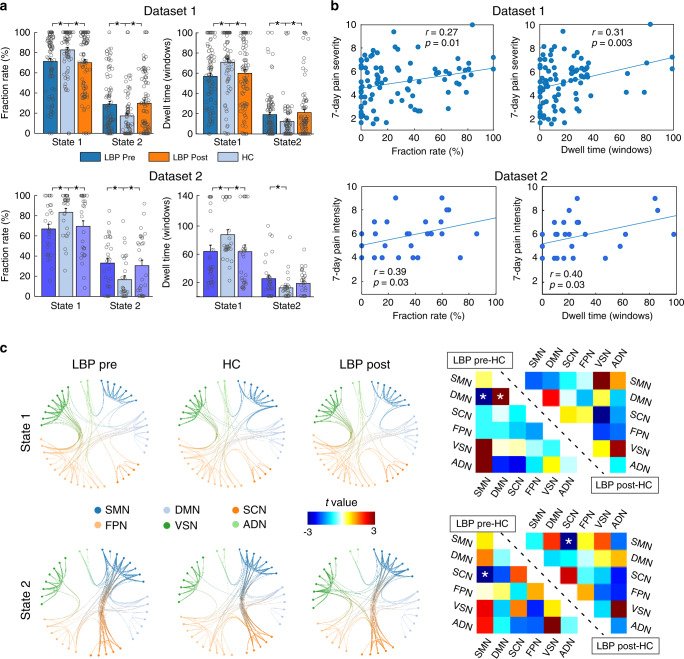
Group differences of occurrences and dFNC patterns in cLBP patients and HCs. **a** In Dataset 1, cLBP patients (pre- and post-maneuver) had significantly lower fraction rate and dwell time in State 1 and higher fraction rate and dwell time in State 2. This finding was validated in Dataset 2. Data are presented as mean values ± SEM. Each circle represents the data for an individual. *N* = 90 and 74 for cLBP patients and HCs in Dataset 1, respectively, and *N* = 30 and 30 for cLBP patients and HCs in Dataset 2, respectively. **b** The fraction rate and dwell time of cLBP patients in State 2 were positively correlated with pain severity/intensity in the past 7 days. Note that pain severity and intensity are two different measures but are typically strongly correlated. **c** cLBP patients and HCs had similar patterns of connectivity in the two states, but they differed in the strength of connections. Asterisks represent significant difference at two-sided *p*_FDR_ < 0.05 for *t*-test. SMN sensorimotor network, DMN default mode network, SCN subcortical network, FPN frontoparietal network, VSN visual network, ADN auditory network. Source data are provided as a Source Data file.

The group differences in occurrences (fraction rate: the proportion of time spent in each state; dwell time: how long the participant stayed in a certain state) are shown in Fig. 3a and Table 2. In Dataset 1, compared with HCs, patients with cLBP had significantly lower fraction rate and dwell time in State 1 but significantly higher fraction rate and dwell time in State 2 in both low-pain (pre-maneuver) and exacerbated-pain (post-maneuver) conditions (*p* < 0.05 false discovery rate [FDR] corrected). We did not find any significant differences in fraction rate and dwell time between low-pain and exacerbated-pain conditions. The significant differences of fraction rate and dwell time between cLBP patients (in both Session 1 and Session 2) and HCs were also observed in Dataset 2 (*p* < 0.05 FDR corrected; the dwell time of cLBP patients in Session 2 was not significantly higher than that of HCs, *p* = 0.17), and the significant differences were further validated using the Yeo parcellation strategy (Supplementary Fig. 10). Overall, these changes suggest that in cLBP patients, the stability of the weaker within-network dFNC (State 1) was significantly affected, while the expression of the stronger between-network dFNC (State 2) was proportionally increased.

**Table 2:** Fraction rate and dwell time (mean ± SEM) for cLBP patients and HCs in two datasets.

Dataset 1			Dataset 2		
	Fraction rate (%)	Dwell time (wins)		Fraction rate (%)	Dwell time (wins)
State 1			State 1		
cLBP pre	71 ± 3	57 ± 3	cLBP sess 1	67 ± 5	63 ± 9
cLBP post	70 ± 3	71 ± 3	cLBP sess 2	69 ± 5	63 ± 9
HC	83 ± 3	60 ± 3	HC	83 ± 4	86 ± 7
State 2			State 2		
cLBP pre	29 ± 3	19 ± 2	cLBP sess 1	33 ± 5	25 ± 5
cLBP post	30 ± 3	21 ± 3	cLBP sess 2	31 ± 5	19 ± 3
HC	17 ± 3	12 ± 2	HC	17 ± 4	13 ± 3

In a further analysis of relationships between dFNC and clinical symptoms in cLBP patients, we found that the fraction rate and dwell time of State 2 were significantly correlated with pain severity/intensity of cLBP patients in the past 7 days (Fig. 3b), indicating that more severe cLBP may result in a higher occurrence rate of State 2.

Figure 3c shows group-specific dFNC profiles (top 100 connections) for cLBP patients before and after the maneuver, as well as HCs, in two different states. In general, patients and HCs had similar patterns of connectivity in the two states (Fig. 3c, left panel). In State 1, before performing the maneuver, cLBP patients had significantly lower connectivity between the SMN and DMN and higher connectivity within the DMN compared with HCs (Fig. 3c, top right panel). In State 2, before and after performing the maneuver, cLBP patients had significantly lower connectivity between the SCN and SMN in State 2 compared with HCs (Fig. 3c, bottom right panel).

### Dynamic network efficiency

**Fig. 4:**
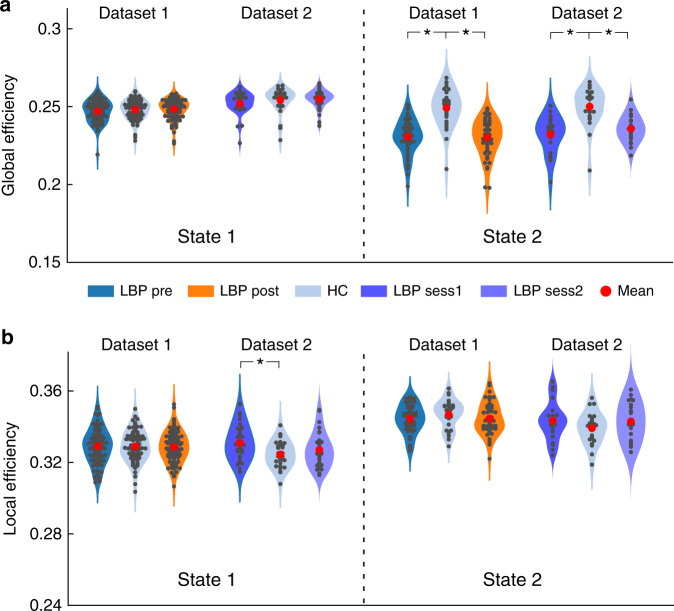
Topologic measures of dFNC states. Global (**a**) and local efficiencies (**b**) in the two different states are shown using violin plots for different groups (pre-maneuver, post-maneuver, and HCs in Dataset 1, *N* = 90 and 74 for cLBP patients and HCs, respectively; LBP session 1, session 2, and HCs in Dataset 2, *N* = 30 and 30 for cLBP patients and HCs, respectively). Each dark dot represents an individual’s value and each red dot indicates the group mean. Asterisks represent significant difference at two-sided *p*_FDR_ < 0.05 for *t*-test. Source data are provided as a Source Data file.

To investigate the topologic organizations of the dFNC states and compare them between groups (pre-maneuver, post-maneuver, and HCs), we applied a graph-theory analysis. Two established and widely validated measures, global and local network efficiencies, were employed to evaluate local and global information transfer in functional brain networks^38^. Figure 4 shows the mean and bootstrapped 95% confidence intervals as well as smoothed density histograms for the global efficiency and local efficiency in each group. In both Dataset 1 and Dataset 2, we observed that cLBP patients exhibited significantly lower global efficiency than HCs in State 2 (*p* < 0.05 FDR corrected; two sample *t*-test), suggesting that the average parallel information transfer in the brain networks of State 2 was less efficient in patients with chronic pain. However, the global efficiency of State 1 was not significantly different in cLBP compared with HCs (*p* > 0.05 for all comparisons in State 1 for the two datasets). In contrast to the findings of global efficiency deficits in cLBP patients, the local efficiency, measuring the average efficiency between critical nodes within a neighborhood, was less affected (we found that cLBP patients in Dataset 2 had statistically higher local efficiency in State 1, but this finding was not validated in Dataset 1). Interestingly, direct comparisons between patients before and after the maneuver did not show a significant difference of global and local efficiencies of dFNC in the two states, indicating that temporary pain exacerbation may not affect efficiencies of brain networks in cLBP patients.

### Abnormal thalamocortical networks

**Fig. 5:**
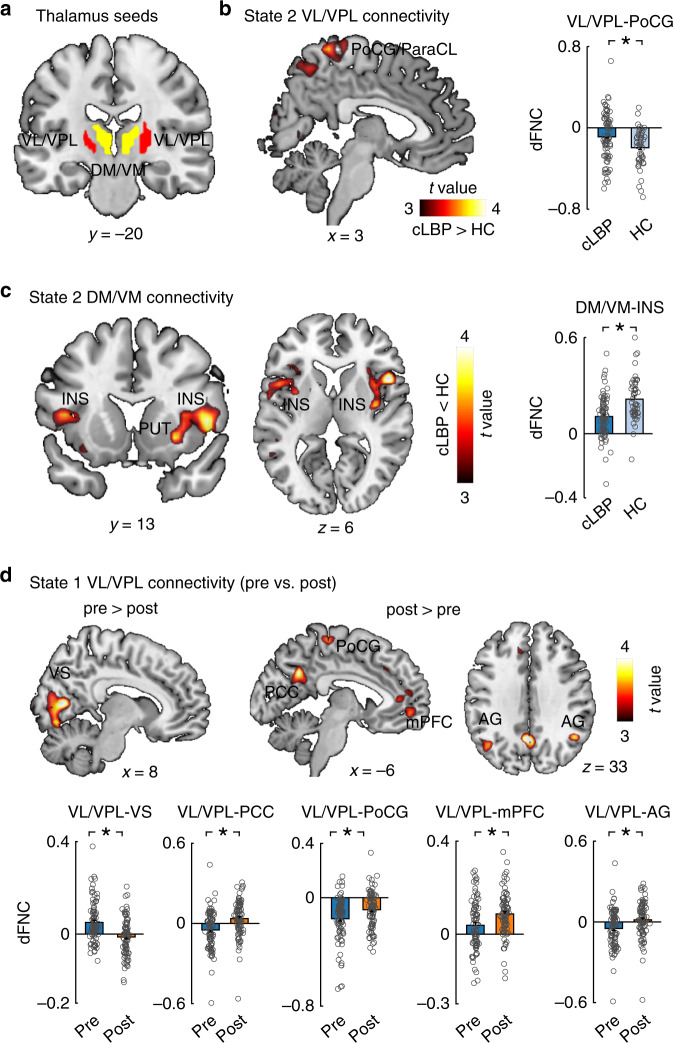
Group differences of thalamus-whole-brain dFNC in two dynamic states. **a** Two seeds, including the ventral lateral/posterolateral (VL/VPL) nucleus and dorsal/ventral medial (DM/VM) nucleus were identified by GICA and used in seed-based correlation analysis. **b** In State 2, cLBP patients had higher dFNC between the VL/VPL and postcentral gyrus (PCoG)/paracentral lobule (ParaCL) and **c** lower dFNC between the DM/VM and insula (INS). **d** In State 1, patients had weaker dFNC between the VL/VPL and visual cortex but stronger dFNC between the VL/VPL and PoCG and typical regions (i.e., medial prefrontal cortex [mPFC], posterior cingulate cortex [PCC], and angular gyrus [AG]) of the DMN. Data are presented as mean values ± SEM (*N* = 90 and 74 for cLBP patients and HCs, respectively). We performed two sample *t*-test for panels **b** and **c**, and paired sample *t*-test for panel **d**, and reported two-sided *p* values. Statistical maps were thresholded at *p* < 0.005 at voxel level and *p*_FDR_ < 0.05 at the cluster level. Comparisons of bars in panel D were corrected for multiple comparisons using FDR. Asterisks represent significant differences at two-sided *p*_FDR_ < 0.05. Source data are provided as a Source Data file.

The findings in the previous sections indicate that State 2 may be a critical dynamic state underlying cLBP pathophysiology. Patients had abnormal dFNC in State 2, particularly between the SCN and SMN. The SCN in our study included the thalamus, putamen, and caudate. Given the important role of the thalamus in the pathophysiology of chronic pain and our hypothesis of abnormal thalamocortical networks in cLBP, we used the thalamus as a seed to investigate thalamus-to-whole-brain dFNC at each state and compared them between groups. In GICA, we identified two ICNs for thalamic nuclei and determined their anatomical location in the thalamus using the MNI coordinates and the BrainNavigator atlas (http://www.thehumanbrain.info/brain/brain_navigator.php) (see “Methods” for details). We found that these two ICNs included the ventral lateral/posterolateral (VL/VPL) nucleus and dorsal/ventral medial (DM/VM) nucleus (Fig. 5a), which have been widely studied in experimental and clinical pain settings. The VL, especially the VPL, is the principal somatosensory nucleus of the thalamus and has been found to be abnormal in humans^39,40^ and rodents^41^. The DM/VM plays a critical role in cognitive functions^42^ and has been found to be dysregulated in migraine^43^. The nucleus-based whole-brain dFNC was estimated using seed-based correlation analysis between the time courses of these two ICNs and time courses of other voxels in the brain in State 1 and State 2. In addition, we also performed conventional static seed-to-voxel connectivity analysis using VL/VPL and DM/VM as seeds.

Figure 5b, c show abnormal VL/VPL and DM/VM-based whole-brain dFNC of cLBP patients in State 2. Compared with HCs, patients had significantly higher connectivity between the VL/VPL and PoCG/ParaCL and lower connectivity between the DM/VM and bilateral insula (the statistical maps were set at a threshold of *p* < 0.005 at voxel level and *p*_FDR_ < 0.05 at the cluster level). These results support our hypothesis that abnormal thalamocortical networks may underlie chronic pain pathophysiology in State 2.

Direct comparisons of VL/VPL and DM/VM-based whole-brain dFNC between pre- and post-maneuver conditions showed that cLBP patients had significantly decreased connectivity between the VL/VPL and visual cortex (i.e., the cuneus, calcarine, and lingual gyrus) but had increased connectivity between the VL/VPL and PoCG and DMN (i.e., medial prefrontal cortex, posterior cingulate cortex, and bilateral angular gyrus) in State 1 (Fig. 5d). Therefore, temporal pain exacerbation may mainly affect thalamocortical networks in the frequent but sparsely connected State 1.

Using conventional static seed-to voxel connectivity, we only found significant differences in DM/VM-based connectivity between cLBP and HCs (Supplementary Fig. 11), but not between pre- and post-maneuver conditions. We did not find any significant differences of VL/VPL-based connectivity in any comparisons.

Considering the complex functions of the thalamus, thalamocortical connections from other nuclei may also account for the connectivity difference between cLBP and HCs as well as between pre- and post-maneuver conditions. Therefore, we performed an exploratory analysis using the same method with the sub-thalamic nuclei, which subdivides the thalamus based on structural connectivity (estimated using probabilistic diffusion tractography) into the following seven large cortical areas: primary motor, somatosensory, occipital, prefrontal, pre-motor, parietal, and temporal (https://fsl.fmrib.ox.ac.uk/fsl/fslwiki/Atlases)^44^. The findings from the sub-thalamic atlas were consistent with those using ICA-derived nuclei, but we also found thalamocortical connectivity differences from other nuclei (Supplementary Note 3 and Supplementary Fig. 12).

In Fig. 5c, we also observed abnormal connectivity between the DM/VM and putamen. Since the basal ganglia (BG) structures (e.g., putamen and caudate) receive input from the thalamus and thalamo-cortico-BG loops play an important role in pain processing^45^, we performed an exploratory analysis using the same method with the putamen and caudate as seeds (identified from GICA). We observed decreased connectivity between the putamen and several brain regions within emotional/affective (e.g., amygdala, hippocampus) and cognitive networks (e.g., anterior cingulate cortex, dorsal lateral prefrontal cortex) in cLBP patients (Supplementary Figs. 13 and 14).

### Cross-site validation

To further validate our findings, and more importantly, to account for the potential confounding effect of vigilance on dFNC^46^, we performed cross-site validation on Dataset 3 with cLBP and HCs. These groups performed an MSIT^24^ before and after the MRI scan and showed similar attention/vigilance levels (see “Methods” for the detailed experimental design of MSIT).

**Fig. 6:**
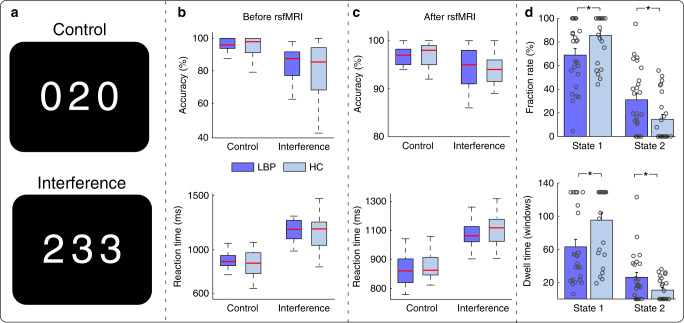
MSIT behavior performance and dFNC occurrences. **a** MSIT trial examples. **b** Accuracy and reaction time for control and interference tasks before resting-state fMRI (rsfMRI) in cLBP and HCs. **c** Accuracy and reaction time for control and interference tasks after rsfMRI in cLBP and HCs. **d** Group differences of occurrences in cLBP and HCs. *N* = 25 and 25 for cLBP patients and HCs, respectively. The boxes in **b** and **c** represent the 25th and 75th percentiles, and the red line represents the median of scores for accuracy or reaction time. The bars and errorbars in **d** represent the mean and SEM. Each circle represents the data for an individual. Asterisks represent significant differences at two-sided *p*_FDR_ < 0.05 for *t*-test. Source data are provided as a Source Data file.

In summary, we found that cLBP patients and HCs performed better (higher accuracy (Acc) and shorter reaction time (RT)) in control trials (e.g., identify “2” in Fig. 6a upper panel) compared with interference trials (e.g., identify “2” in Fig. 6a lower panel), but the task performance did not differ between groups (Fig. 6b, c). Although we were not able to experimentally track vigilance level during the resting-state fMRI scan, the MSIT (particularly the control condition of the task) provided evidence that cLBP patients and HCs had similar vigilance/attention levels both before and after the fMRI scan. In this cohort, we were able to replicate the findings obtained from Datasets 1 and 2 [i.e., cLBP patients spent longer time (larger fraction rate and dwell time) in State 2 compared with HCs (Fig. 6d)].

### Quality control analyses

To further explore the potential effects of vigilance/drowsiness on our results in Datasets 1 and 2, we compared the putative vigilance levels between cLBP patients and HCs using an fMRI-based vigilance spatial template^47^ and fMRI global signal amplitudes^48^. We did not find that vigilance levels differed between the two cohorts or between the two dFNC states (Supplementary Note 4).

In addition, similar to our previous study, we extracted FC features based on the well-established rsFC neural markers of drowsiness^49^ and found that these features could significantly classify cLBP patients and HCs, as well as the two dFNC states. These results suggest that there was no systematic difference in vigilance/drowsiness levels that may have confounded our findings. Details of the analysis and results are provided in Supplementary Note 4 and Supplementary Fig. 15.

To rule out the potential effects of head motion and opioid usage on our results, we performed quality control analyses. We first found that head motion [as represented by the maximal frame displacement value^50^] did not differ between groups and did not show correlations with fraction rate, dwell time, or pain severity (Supplementary Note 5 and Supplementary Fig. 16). Second, only five cLBP patients in Dataset 1 and one cLBP patient in Dataset 2 reported that they took opioid medications before the scans. Given the sample sizes of the two datasets, we thus believe that our results were not affected by opioid usage.

## Discussion

Given the dynamic and condition-dependent nature (e.g., mental states, diseases) of brain functional connectivity, even during “rest,” time-varying characteristics of rsFC may reveal neural mechanisms/pathophysiology that cannot be discovered through static rsFC alone. In the present study, we identified two reoccurring dFNC states characterized by different connectivity patterns. Of these two dFNC states, the less frequent state, characterized by strong negative connectivity between the SCN and SMN, is associated with the following characteristics: (1) the occurrences of this state were significantly higher in cLBP patients and correlated with pain severity, (2) patients exhibited significantly lower efficiency of information transfer in functional brain networks in this state, and (3) patients had abnormal thalamocortical networks in this state. In contrast, the altered clinical pain intensity was associated with thalamocortical networks in the other more frequent state, which was characterized by sparsely connected within-network dFNC.

Recent studies have shown the neuronal origins of dynamic rsFC^51^ and have suggested that temporal organization of dynamic rsFC patterns follow specific sequential orders in awake rats and humans^52^. Therefore, it is believed that rsFC brain networks may have several reoccurring states throughout the entire fMRI scan^36^. In this study, we found that both patients and HCs had two dFNC states. State 1, the more frequent and sparsely connected state, was characterized by within-network connectivity and was similar to sFNC patterns. A dFNC state that resembles sFNC patterns typically accounts for the largest percentage of windows and time^14,18,36,37,51,53^. It is speculated that such a weak and diffused state represents the average of a large number of additional states with less variability^36,54^ and may be associated with self-referential processing and even drowsiness^16,55^. This state may be considered a steadier state and signifies the average of less variable rsFC, thereby sharing similar connectivity patterns with sFNC.

State 2 was a less frequent but more strongly interconnected state that was represented by negative connectivity between the SCN and SMN. Patients tended to spend more time in this state, and the state’s occurrence showed significant correlation with pain severity. We speculated that the increased occurrence of State 2 for cLBP patients may be due to abnormal cortical-subcortical interaction^56^, and this was confirmed by our subsequent analyses (i.e., topological analysis and thalamocortical network analysis). Interestingly, a similar dFNC state was identified in previous studies on healthy and diseased populations, including those with schizophrenia^17^, migraine^15^, bipolar disorder^53^, and Parkinson’s disease^37^. Although these diseases have different pathophysiologies, they share a common TCD model^6,8^. The dFNC patterns in this state were always characterized by connectivity between the SCN and other disease-related networks; for example, between the SCN and VSN in migraine^15^, between the SCN and SMN/VSN in schizophrenia^14^, and between the SCN and SMN in chronic pain in the present study. Several studies have shown that the temporal properties and dFNC patterns in this state have associations with clinical symptoms that cannot be observed between sFNC and the same clinical scores^54,57^. Thus, this transient dFNC state may be better for revealing a disease’s pathophysiology by excluding non-relevant steady dFNC states (e.g., State 1 in this study).

The importance of State 2 in revealing cLBP pathophysiology was further supported by reduced efficiency of global information transfer in functional brain networks. Previous studies have found global dysfunction of multisensory information processing and integration in chronic pain patients^58,59^. In our study, we applied topologic measures for examining the global and local efficiencies and provided direct evidence of disrupted functional segregation and integration in brain networks of State 2 but not in State 1. The loss of brain efficiency in chronic pain patients in State 2 may lead to more occurrences/time spent in this state.

Evidence from electrophysiological studies has shown aberrant intrinsic electrical activity in the thalamocortical loop and suggests that the TCD^6^ underlies multisensory dysfunction in chronic NP^7–9^. fMRI evidence also suggests that NP is associated with disturbed thalamocortical activity^10^. We demonstrated that cLBP patients, compared with HCs, had disrupted connectivity between the VL nucleus and PoCG/ParaCL, as well as between the DM and insula. Interestingly, we found that the abnormalities were only observed in State 2, not in State 1. The VL, especially the VPL nucleus, is the principal somatosensory nucleus of the thalamus, which sends projections to PoCG and ParaCL for somatosensation. The dysfunction of the VL/VPL and its projections have been observed in experimentally induced pain in humans (e.g., cold, laser stimuli)^39,40^ and chronic pain in rats^41^. On the other hand, the DM plays a role in cognitive functions together with other cortical brain areas (e.g., anterior cingulate, dorsolateral prefrontal cortex, and insula)^60^ and is involved in the cognitive deficits of several neurological and psychiatric disorders^42^. These findings suggest abnormal thalamocortical networks of both sensory and cognitive domains in cLBP patients, which is consistent with studies demonstrating that chronic pain may modulate brain functions beyond the pain/somatosensory system itself^21–23^.

Previous studies investigating resting-state brain activity/connectivity in chronic pain patients normally lack any concomitant experimental manipulation and therefore may mix abnormalities stemming from intrinsic brain dysfunction and those stemming from the intensity of clinical pain. To better understand the specific relationships between chronic pain and thalamocortical networks, we experimentally manipulated levels of spontaneous LBP on patients to create two conditions: a low back pain and a high back pain condition. Our results provide direct evidence that increasing spontaneous back pain only alters thalamocortical networks (between the VL/VPL nucleus and PoCG/DMN) in State 1 but not in State 2. Taken together, our results suggest that thalamocortical networks, which underlie chronic pain pathophysiology, may be reflected in State 2, while clinical pain may modulate thalamocortical networks in State 1.

It is worth mentioning that our exploratory analysis with the putamen as a seed extended the findings from thalamocortical networks to thalamo-cortico-BG networks. The altered function of thalamo-cortico-BG loops in chronic pain has been reported previously^45,56^ and may result in altered integration of sensorimotor responses, cognitive impairment, and emotional processing.

There are several limitations to this study. First, although we have performed substantial quality control analyses to rule out the potential effect of vigilance differences between cLBP patients and HCs, and we have included an independent dataset to validate our findings, future studies could include EEG, cardiac, respiratory, or eye-tracking data to monitor vigilance level during scans. Second, our present study focuses on thalamus-related sensory system dysfunction in chronic pain (e.g., using maneuvers to increase pain levels). Future studies with specific experimental designs and hypotheses are needed to explore the abnormal brain dynamics in associative and limbic systems. Third, clinical utility was not explored in this study. Because patients from the three cohorts exhibited consistent abnormalities in rsFC time-varying characteristics and thalamocortical networks, it is plausible that they can be indicators/markers of evaluating treatment effects, especially for those approaches targeting thalamocortical networks (e.g., brain stimulation)^61^.

## Methods

### Participants

The present study included three independent datasets with a total of 274 participants. The first dataset (Dataset 1) included 90 patients diagnosed with cLBP with a duration of at least 6 months confirmed by a clinical evaluation and 74 matched HCs. The second dataset (Dataset 2) included 30 cLBP and 30 HCs. The third dataset (cross-site validation) included 25 cLBP and 25 HCs. All patients in the three datasets met the same inclusion criteria and had no other chronic pain comorbidities. Details of the inclusion criteria can be found in Supplementary Note 6. The Institutional Review Board (IRB) of Massachusetts General Hospital (MGH) approved the first two datasets, and the Research Ethics Committee of the Xian Jiao Tong University approved the third dataset. All experiments were performed in accordance with the guidelines set forth by the IRB for ethics and protection of human participants. All participants gave written consent.

### Experimental procedures

In the first dataset, all cLBP patients underwent two resting-state fMRI scan sessions. After the first MRI session, patients stepped out of the scanner and performed pain-exacerbating maneuvers to increase their LBP so that we could investigate the brain activity/connectivity changes following temporary back pain intensification. The maneuvers were tailored to each patient based on what the patient reported would exacerbate his or her LBP, such as lumbar flexion, extension, or rotation^20,62^. After the maneuvers, which took ~10–15 min, patients entered the scanner for another identical MRI session. All patients were required to rate their pain intensity before and after the two MRI sessions. HCs did not perform maneuvers and underwent only one MRI session.

In the second dataset, cLBP patients did not perform maneuvers, and they underwent two MRI sessions separated by about 2 weeks. HCs underwent only one MRI session.

In the third dataset, cLBP patients and HCs performed a MSIT^24^ before and after the MRI scan to measure and increase their attention level. The attention and cognition components of MSIT are closely related to vigilance/arousal level^63^, and other similar visual attention tasks have been used to study subjects before and after sleep deprivation^64,65^, suggesting that a drowsy state/insufficient sleep may reduce visual attention task performances. The MSIT was presented by e-prime 2.0 (Psychology Software Tools, PA, USA). In brief, subjects were given response boxes (with numbers of 1, 2, and 3) and were required to view sets of three numbers (i.e., 1, 2, 3, 0) in the center of the screen lasting for 1.75 s, with one number always being different from the other two numbers. Subjects were instructed to report, via button press, the number that was different from the other two items. There were two different tasks: a control task in which the distractors were zeros (0) and target numbers were always aligned with the same position as on the response box, and an interference task in which the distractors were other numbers (i.e., 1, 2, or 3) and target numbers were not aligned with the same position as on the response box (Fig. 6a). Two different types of trials appeared alternately (i.e., Control-Interference-Control-Interference) with a total of 96 trials (48 trials for each task; completed in two blocks outside the MRI room) before resting-state MRI and a total of 192 trials (96 trials for each task; completed in four blocks inside the MRI room) after resting-state MRI. Stimulus and inter-stimulus intervals were 1.75 and 0 s, respectively. For all trials, subjects were instructed to answer as quickly and accurately as possible. The RTs and Acc were used as behavioral measures to assess subjects’ attention/vigilance level.

### MRI acquisition

MRI data in the first and second datasets were acquired using a 32-channel radio frequency head coil in a 3-T Siemens scanner at the Martinos Center for Biomedical Imaging. T2-weighted functional data encompassing the whole brain were acquired with gradient-echo planar imaging (repetition time: 3000 ms, echo time: 30 ms, flip angle: 90°, slice thickness: 3 mm, interslice gap: 0.88 mm, FOV: 240 mm, and 44 slices). Subjects in Dataset 1 and Dataset 2 had 6-min and 8-min resting-state fMRI scans, respectively. High-resolution brain structural images were also acquired with a T1-weighted three-dimensional multi-echo magnetization-prepared rapid gradient-echo sequence (repetition time: 2500 ms, echo time: 1.69 ms, slice thickness: 1 mm, flip angle: 7°, FOV: 256 mm, and 176 slices).

The MRI data in the third dataset were acquired at the First Affiliated Hospital of Xian Jiao Tong University using an 8-channel head coil in a 3-T GE scanner (repetition time: 2500 ms, echo time: 30 ms, flip angle: 90°, slice thickness: 3 mm, interslice gap: 0 mm, FOV: 256 mm, and 50 slices). The structural images were acquired with a T1-weighted 3-dimensional fast-spoiled gradient-echo sequence (repetition time: 10.7 ms, echo time: 4.8 ms, slice thickness 1 mm, flip angle: 7°, FOV: 256 mm, and 140 slices).

### fMRI preprocessing

fMRI data were preprocessed using CONN toolbox version 17f (https://www.nitrc.org/projects/conn). The first five scans were removed for signal equilibrium and participants’ adaptation to the scanner’s noise. Preprocessing steps included a standard pipeline (functional realignment and unwarp, functional slice-timing correction, structural segmentation and normalization, functional normalization, functional outlier detection, and functional smoothing with a 5-mm full-width at half-maximum Gaussian kernel). ART (http://www.nitrc.org/projects/artifact_detect/) was also applied to detect motion during the resting-state fMRI scan. Time points in subjects’ images were marked as outliers if the global signal exceeded three standard deviations from the mean or if scan-to-scan motion deviation exceeded 0.5 mm. Those outliers, in addition to the linear and polynomial trends of six head-motion parameters^50^, were included as nuisance regressors during the denoising procedure in the post-processing steps of GICA. Note that we did not perform global signal regression in the preprocessing.

### dFNC analysis

The framework for characterizing dFNC to detect atypical thalamocortical networks in cLBP patients is shown in Supplementary Fig. 17 and in our previous publication^15^. In brief, this framework consisted of four major steps: (1) conduct a GICA with spatial reference ^54^ to decompose whole-brain resting-state fMRI data into multiple ICs and select ICNs from ICs; (2) calculate dFNC among ICNs using a sliding-window approach with graphical LASSO; (3) conduct a *k*-mean clustering on the dFNC estimates to identify distinct states (dFNC states) and the fraction rate/dwell time; and (4) apply graph-theory measures on each dFNC state to demonstrate the efficiency of information transfer in functional brain networks. The technical details pertaining to each step are described in the Supplementary Methods. It is worth mentioning that the global mean signal per time point was removed as the standard step in GICA^55^.

### Abnormal thalamocortical networks in each dFNC state

To explore the abnormal thalamocortical networks, we performed the thalamus-to-whole-brain connectivity analysis within each dFNC state (for VL/VPL nucleus and DM/VM nucleus respectively; identified from GICA; threshold with *t* value > 10 and confined by the AAL thalamus atlas). In addition to BrainNavigator, we also compared our masks to locations of FSL sub-thalamic atlas and Morel atlas^66^. Given the potential limitations of ICA technique in brain parcellation, the nuclei obtained from ICA may cover a relatively larger area compared with parcellations based on cyto- and myelo-architecture information^67^, as well as other noninvasive thalamic parcellations using probabilistic diffusion tractography^44^ and using functional connectivity-based winner-take-all strategy^68^. We therefore defined the nucleus based on its most predominant anatomical location in the thalamus.

Then, we (1) calculated the correlations between the time series of the thalamus and time series of all other voxels in the brain within each sliding window, resulting in a connectivity map (Fisher *z*-transformed) for each window; (2) averaged the connectivity maps within each state (i.e., averaged connectivity maps within all windows corresponding to State 1/State 2); and (3) performed statistical comparisons between different groups (i.e., cLBP patients vs. HCs; pre-maneuver cLBP patients vs. post-maneuver cLBP patients), thresholded at *p* < 0.005 at voxel level and *p*_FDR_ < 0.05 at cluster level.

### Reporting summary

Further information on research design is available in the Nature Research Reporting Summary linked to this article.

## Supplementary information

Supplementary Information

Reporting Summary

## Data Availability

The MRI data are from multiple sources. Not all participants gave their permission to share their data with the public. Some datasets are part of longitudinal studies that will generate more than one manuscript. The data will eventually be made available, with the permission of the participants, once these manuscripts are completed. Reasonable requests can be sent to the corresponding author (J.K.). The data used for deriving the group template of ICNs are available from HCP website (https://www.humanconnectome.org/) and GSP website (https://dataverse.harvard.edu/dataverse/GSP). The group template of ICNs derived from HCP and GSP for spatially constrained ICA can be requested from V.C. (vcalhoun@gsu.edu). Source data are provided with this paper.

## Source data

Source Data

## Code availability

Most MATLAB codes were included in the CONN toolbox (fMRI preprocessing and ART), GIFT toolbox (GICA), and GRETNA toolbox (graphic measures). Other customized codes are available in the github (https://github.com/yihengtu/Dynamic-connectivity-for-fMRI.git).
