# Author Correction: Distinct thalamocortical network dynamics are associated with the pathophysiology of chronic low back pain

**DOI:** 10.1038/s41467-020-18191-4

**Published:** 2020-08-25

**Authors:** Yiheng Tu, Zening Fu, Cuiping Mao, Maryam Falahpour, Randy L. Gollub, Joel Park, Georgia Wilson, Vitaly Napadow, Jessica Gerber, Suk-Tak Chan, Robert R. Edwards, Ted J. Kaptchuk, Thomas Liu, Vince Calhoun, Bruce Rosen, Jian Kong

**Affiliations:** 1 Department of Psychiatry, Massachusetts General Hospital, Harvard Medical School, Charlestown, MA USA; 2 Department of Radiology, Martinos Center for Biomedical Imaging, Massachusetts General Hospital, Harvard Medical School, Charlestown, MA USA; 3 Tri-Institutional Center for Translational Research in Neuroimaging and Data Science (TReNDS), Georgia State University, Georgia Institute of Technology, Emory University, Atlanta, GA USA; 4 Department of Medical Imaging, First Affiliated Hospital of Xi’An Jiao Tong University College of Medicine, Xi’an, Shaan’Xi China; 5 Center for Functional MRI, University of California San Diego, La Jolla, CA USA; 6 Department of Anesthesiology, Perioperative and Pain Medicine, Brigham and Women’s Hospital, Harvard Medical School, Boston, MA USA; 7 Beth Israel Deaconess Medical Center, Harvard Medical School, Boston, MA USA

**Keywords:** Chronic pain, Chronic pain

Correction to: *Nature Communications* 10.1038/s41467-020-17788-z, published online 7 August 2020.

The original version of this Article contained an error in the author affiliation.

Affiliation 4 incorrectly read ‘Department of Medical Imaging, First Affiliated Hospital of Xi’An Jiao Tong University College of Medicine, Xi’an, Shaan’Xi, China’.

This has now been corrected in both the PDF and HTML versions of the Article.

