# Supplementary material for: Distinct thalamocortical network dynamics are associated with the pathophysiology of chronic low back pain
Source: Nat Commun. 2020 Aug 7;11:3948. doi: 10.1038/s41467-020-17788-z (PMC7414843; doi:10.1038/s41467-020-17788-z)
Supplement: Supplementary file 1 — Supplementary Information [file 41467_2020_17788_MOESM1_ESM.pdf]

Supplementary Information for

Distinct thalamocortical network dynamics are associated with the pathophysiology of chronic low back pain

Tu et al.

Supplementary Note 1: Group template of ICNs from Human Connectome Project and Genomics Superstruct Project

The group template of ICNs was derived from healthy populations in Human Connectome Project (HCP) and Genomics Superstruct Project (GSP) with different temporal resolutions (HCP: 0.72 s; GSP: 3 s). HCP and GSP datasets were preprocessed using preprocessing pipelines similar to those introduced in the main text. In total, 1005 subjects were chosen from the GSP dataset and 823 subjects were chosen from the HCP dataset. For each of these independent datasets, principal component analysis (PCA) was performed to reduce the subject-specific data into 110 principal components (PCs) that preserve more than 95% variance of the original data. The 110 principal components of each subject were concatenated across subjects and then reduced to 100 PCs at the group level. The infomax ICA algorithm was conducted to decompose the 100 PCs into 100 independent components (ICs). This procedure was repeated 20 times in ICASSO, in which the best run was selected to ensure estimation stability.

We used the template as a reference within a spatially constrained ICA [1] to compute individual spatial maps and time courses for our datasets. Considering the relevance of brain regions and networks to chronic pain, we selected 45 intrinsic connectivity networks (ICNs) from 100 ICs and categorized them into 6 functional networks (Supplementary Figures 1-6 and Table 1).

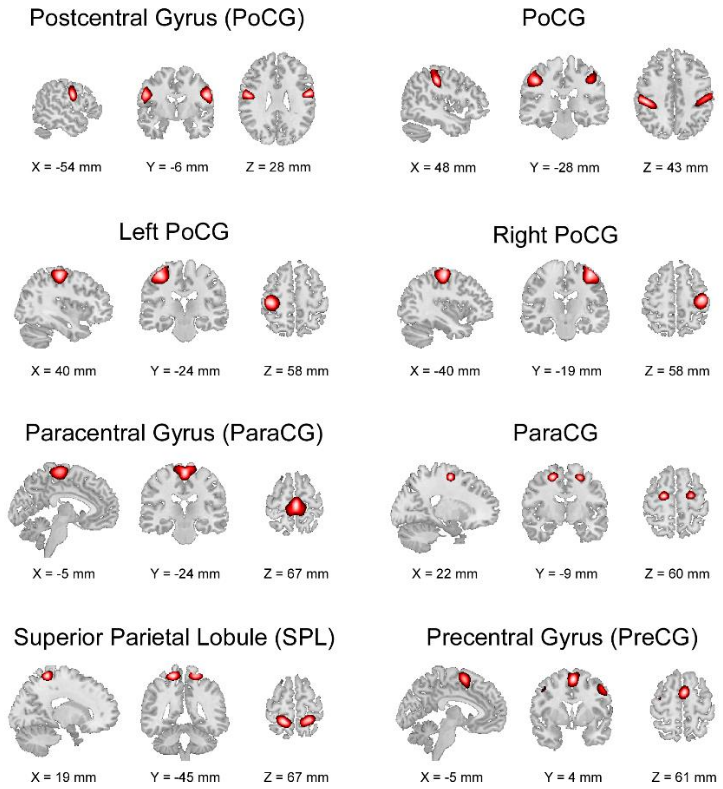

Supplementary Figure 1. Spatial maps (SMs) of intrinsic connectivity networks (ICNs) in the sensorimotor network. ICNs were thresholded at $|t|>3$ for visualization, where a one-sample t-test was computed across single-subject SMs.

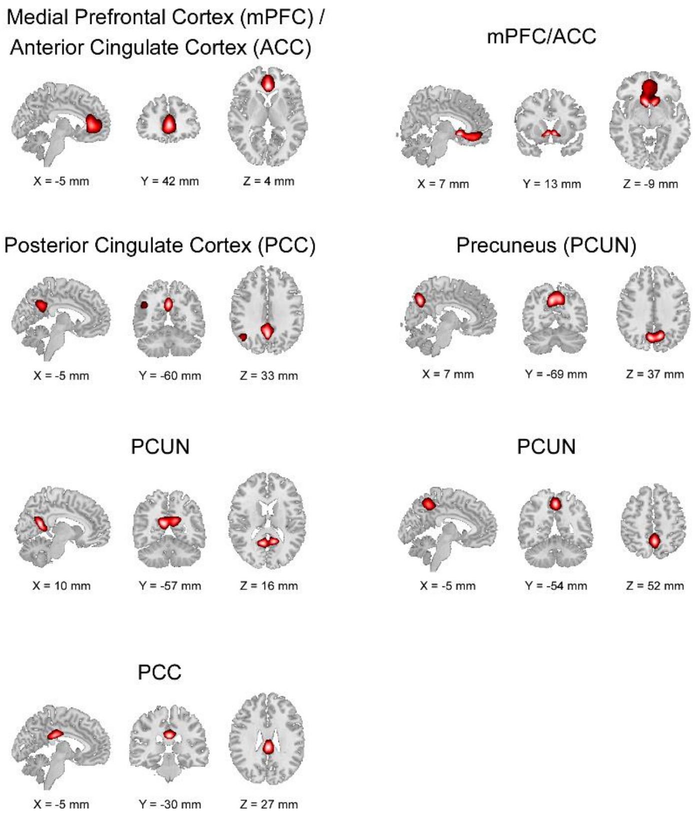

Supplementary Figure 2. Spatial maps (SMs) of intrinsic connectivity networks (ICNs) in the default mode network. ICNs were thresholded at |t|>3 for visualization, where a one-sample t-test was computed across single-subject SMs.

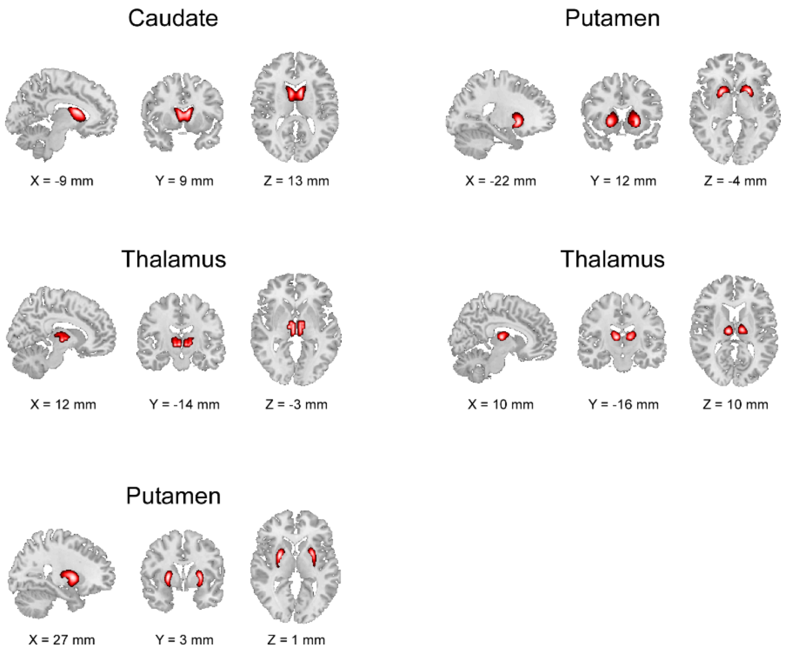

Supplementary Figure 3. Spatial maps (SMs) of intrinsic connectivity networks (ICNs) in the subcortical network. ICNs were thresholded at |t|>3 for visualization, where a one-sample t-test was computed across single-subject SMs.

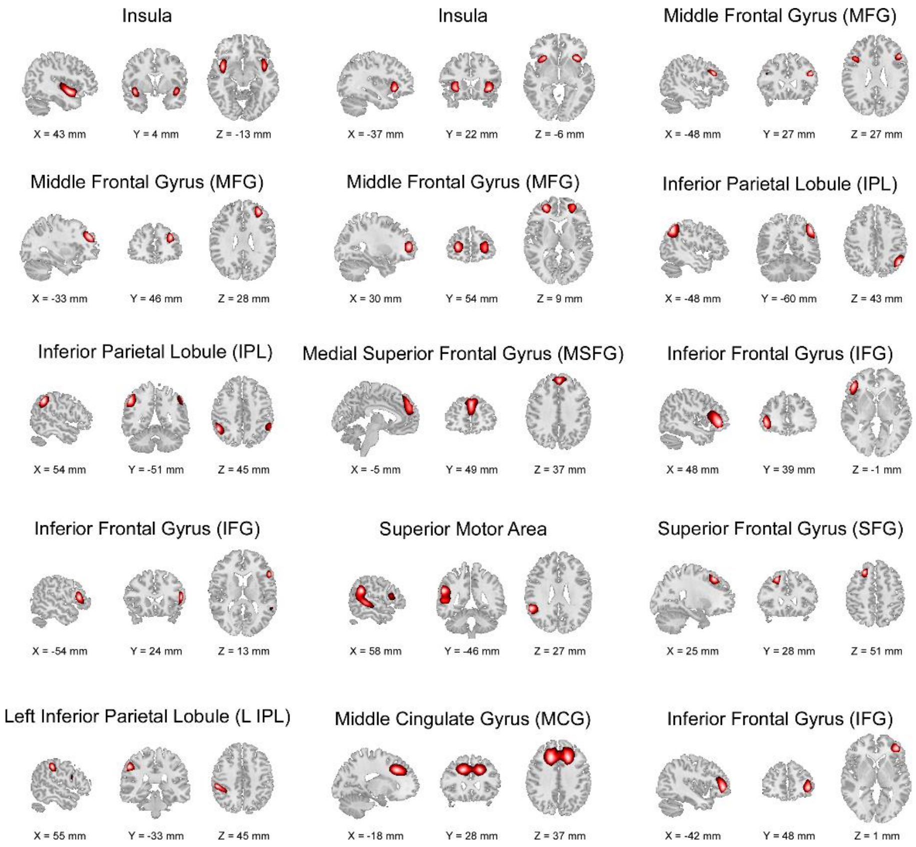

Supplementary Figure 4. Spatial maps (SMs) of intrinsic connectivity networks (ICNs) in the fronto-parietal network. ICNs were thresholded at |t|>3 for visualization, where a one-sample t-test was computed across single-subject SMs.

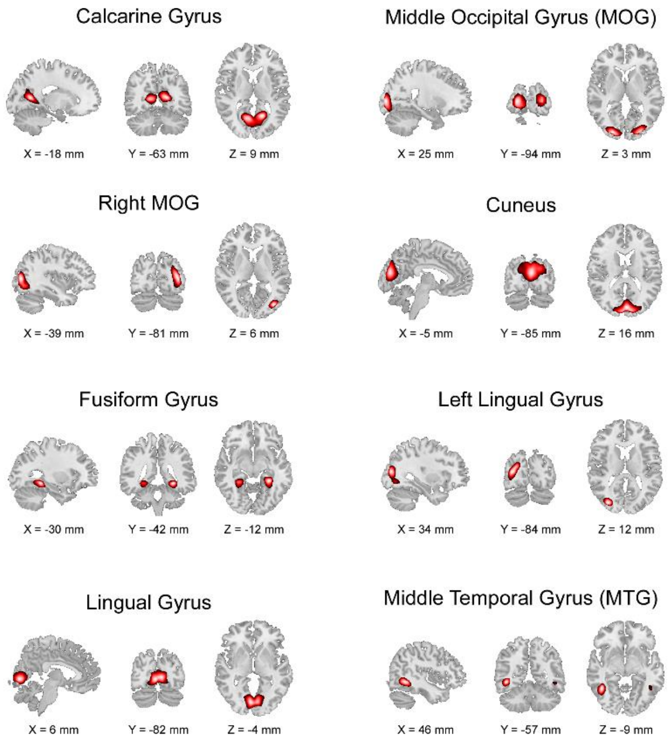

Supplementary Figure 5. Spatial maps (SMs) of intrinsic connectivity networks (ICNs) in the visual network. ICNs were thresholded at |t|>3, where a one-sample t-test was computed across single-subject SMs.

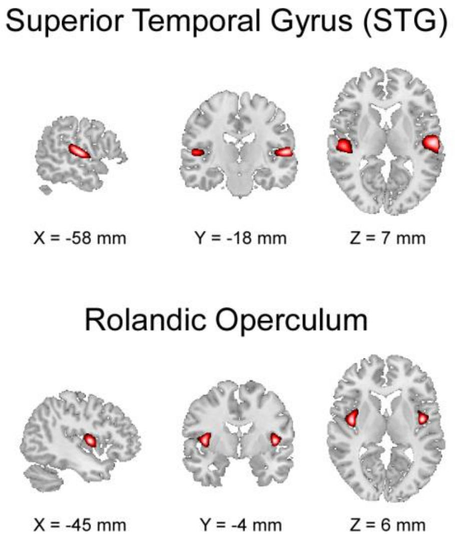

Supplementary Figure 6. Spatial maps (SMs) of intrinsic connectivity networks (ICNs) in the auditory network. ICNs were thresholded at |t|>3, where a one-sample t-test was computed across single-subject SMs.

Supplementary Table 1: Peak Coordinates of Intrinsic Connectivity Networks (ICNs)

ICNs	X	Y	Z
**Sub-cortical network (SCN)**			
Caudate (69)	8.5	8.5	12.5
Dorsal medial thalamus (53)	-2.5	-12.5	2.5
Putamen (98)	-26.5	2.5	0.5
Caudate (99)	21.5	11.5	-3.5
Ventral lateral thalamus (45)	-9.5	-15.5	9.5
**Auditory network (ADN)**			
Superior temporal gyrus ([STG], 21)	57.5	-17.5	6.5
Rolandic operculum ([Rolandic], 56)	44.5	-3.5	5.5
**Sensorimotor network (SMN)**			
Postcentral gyrus ([PoCG], 3)	53.5	-5.5	27.5
Left postcentral gyrus ([L PoCG], 9)	-39.5	-23.5	57.5
Paracentral lobule ([ParaCL], 2)	0.5	-23.5	66.5
Right postcentral gyrus ([R PoCG], 11)	39.5	-18.5	57.5
Superior parietal lobule ([SPL], 27)	-18.5	-44.5	66.5
Paracentral lobule ([ParaCL], 54)	-21.5	-8.5	59.5
Precentral gyrus ([PreCG], 66)	0.5	3.5	60.5
Postcentral gyrus ([PoCG], 72)	-47.5	-27.5	42.5
**Visual network (VSN)**			
Calcarine gyrus ([CalcarineG], 16)	17.5	-62.5	8.5
Middle occipital gyrus ([MOG], 5)	-24.5	-93.5	2.5
Cuneus (15)	2.5	-84.5	15.5
Right middle occipital gyrus ([R MOG], 12)	38.5	-80.5	5.5
Fusiform gyrus (93)	29.5	-41.5	-11.5
Inferior occipital gyrus ([IOG], 20)	-33.5	-83.5	11.5
Lingual gyrus ([LingualG], 8)	-5.5	-81.5	-3.5
Middle temporal gyrus ([MTG], 77)	-45.5	-56.5	-8.5
**Fronto-parietal network (FPN)**			
Inferior parietal lobule ([IPL], 68)	47.5	-59.5	42.5
Insula (33)	36.5	21.5	-5.5
Superior medial frontal gyrus ([SMFG], 43)	-0.5	48.5	36.5
Inferior frontal gyrus ([IFG], 70)	-47.5	38.5	-0.5
Right inferior frontal gyrus ([R IFG], 61)	53.5	23.5	12.5
Middle frontal gyrus ([MiFG], 55)	47.5	26.5	26.5
Inferior parietal lobule ([IPL], 63)	-53.5	-50.5	44.5
Superior motor area ([Superior MA], 84)	-57.5	-45.5	26.5
Superior frontal gyrus ([SFG], 96)	-24.5	27.5	50.5
Middle frontal gyrus ([MiFG], 88)	32.5	45.5	27.5
Left inferior parietal lobule ([L IPL], 81)	-54.5	-32.5	44.5
Middle cingulate cortex ([MCC], 37)	17.5	27.5	36.5
Inferior frontal gyrus ([IFG], 67)	41.5	47.5	0.5
Middle frontal gyrus ([MiFG], 38)	-29.5	53.5	8.5
Middle cingulate cortex ([MCC], 91)	2.5	-3.5	48.5
**Default-mode network (DMN)**			
Precuneus (32)	-6.5	-68.5	36.5
Precuneus (40)	-9.5	-56.5	15.5
Anterior cingulate cortex/medial prefrontal cortex ([ACC/mPFC], 23)	0.5	41.5	3.5
Posterior cingulate cortex ([PCC], 71)	0.5	-29.5	26.5
Anterior cingulate cortex/medial prefrontal cortex ([ACC/mPFC], 17)	-6.5	12.5	-8.5
Precuneus (51)	0.5	-53.5	51.5
Posterior cingulate cortex ([PCC], 94)	-0.5	-59.5	32.5

Supplementary Note 2: Brain parcellation using Yeo atlas

To increase the validity of our study, we tested an additional parcellation strategy of using the 7-network functional atlas by Yeo and colleagues [2]. Details of the parcellation strategy can be found at https://github.com/ThomasYeoLab/CBIG/tree/master/stable_projects/brain_parcellation/ Yeo2011_fcMRI_clustering. We added 6 regions in the subcortical network (bilateral thalamus, bilateral caudate, and bilateral putamen) and 6 regions in the sensorimotor network (bilateral postcentral gyrus, bilateral precentral gyrus, and bilateral paracentral lobule) from AAL atlas to match the regions identified from group independent component analysis, resulting in a parcellation of 61 regions and 8 networks (Supplementary Figure 7).

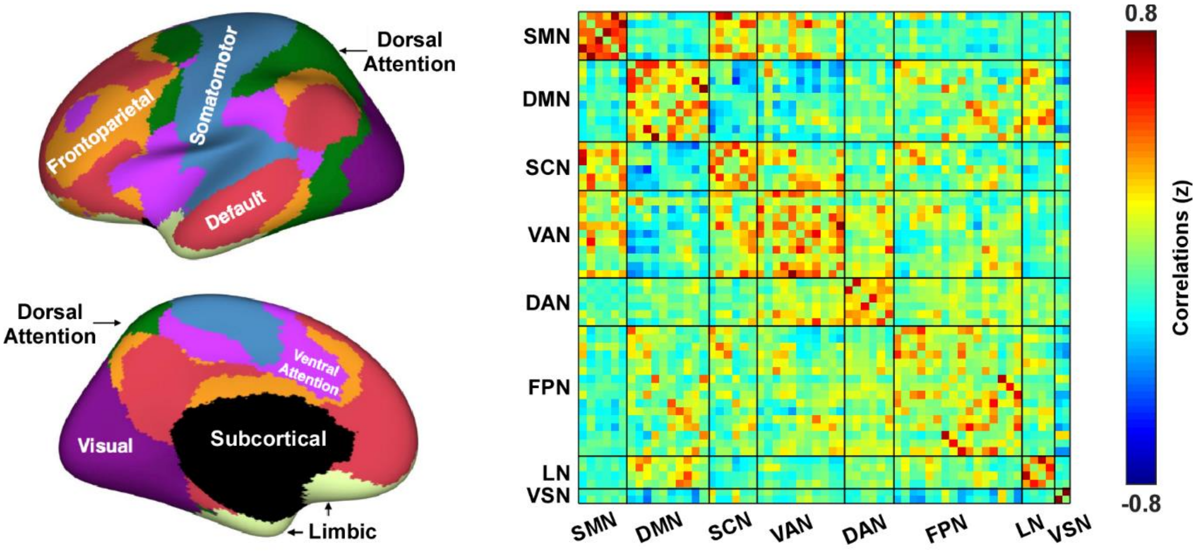

Supplementary Figure 7. Brain parcellation using Yeo functional atlas and static functional connectivity matrix.

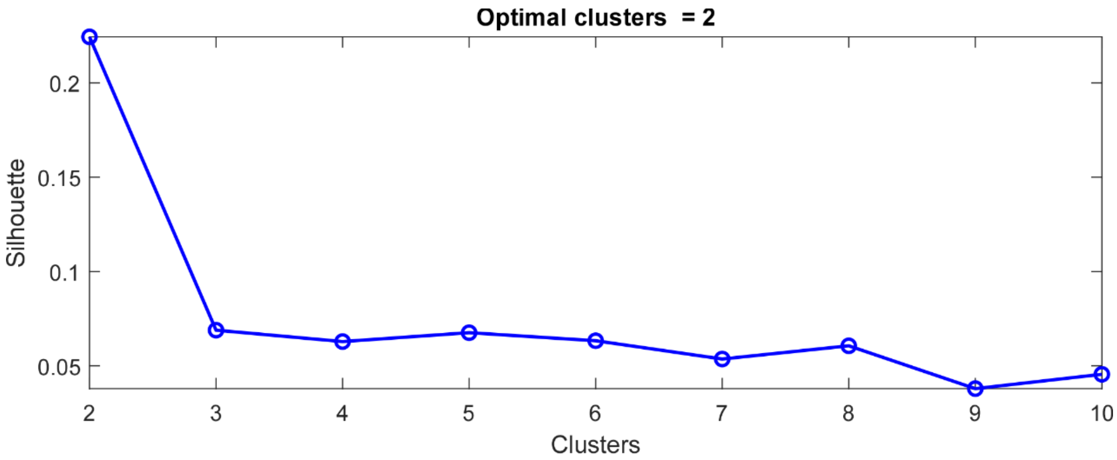

Supplementary Figure 8. Determining the optimal number of clusters varying from 2-10. The Silhouette method was used, and the optimal number was 2.

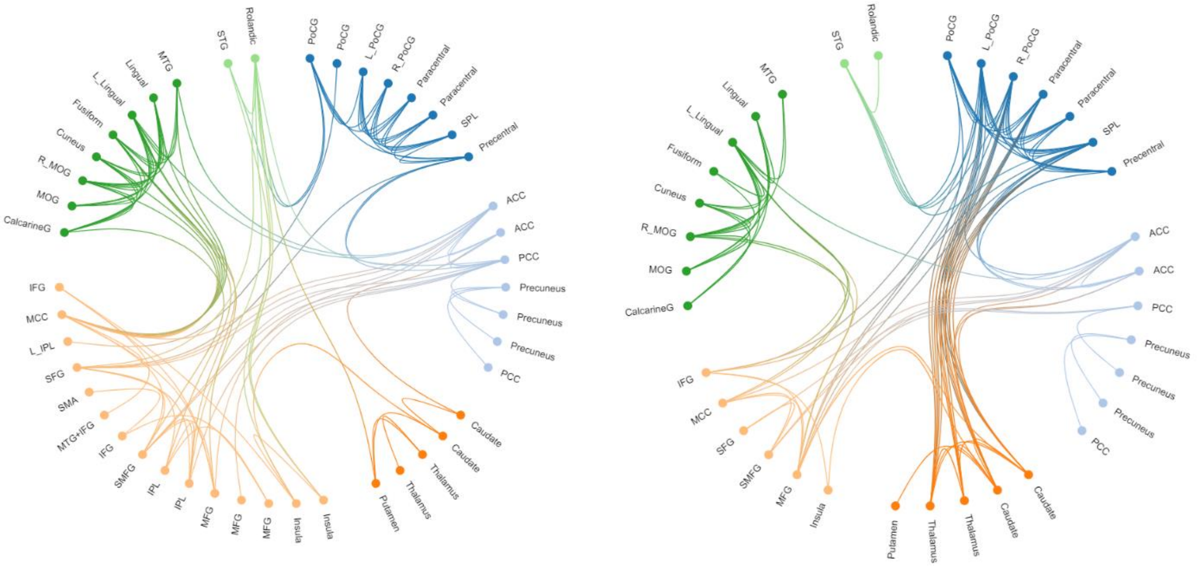

Supplementary Figure 9. Details of top 100 connections (as indexed by the strength of connectivity) in State 1 (left panel) and State 2 (right panel) with labels of ICNs.

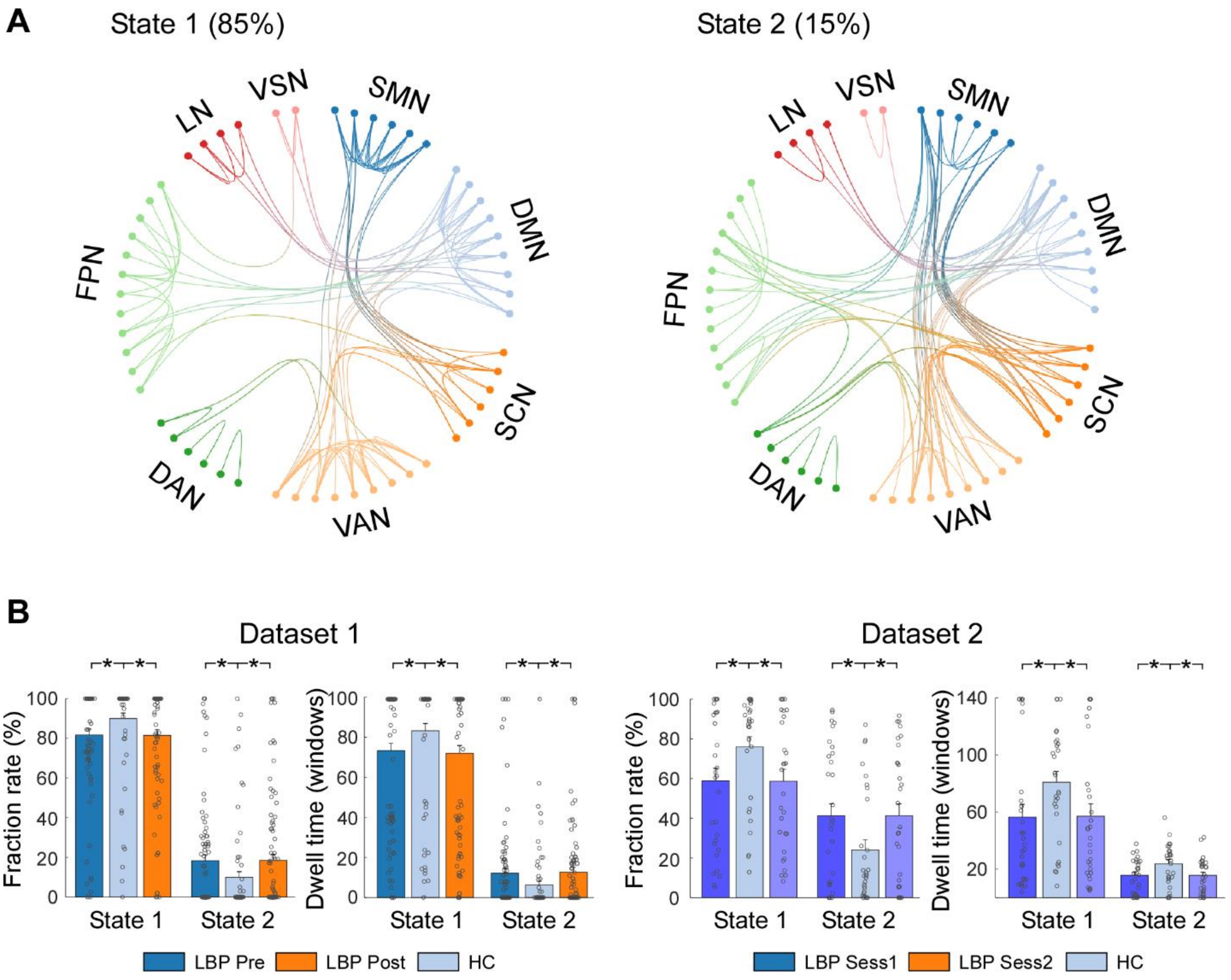

Supplementary Figure 10. Findings of clustering analysis based on Yeo parcellation. A. Top 100 of the functional connectivity in two states, a more frequent (85% of total occurrences) and sparsely connected State 1 and a less frequent (15% of total occurrences) and more strongly interconnected State 2. In State 2, we observed strong connectivity between SCN and SMN. B. Compared to HCs, patients with cLBP had significantly lower fraction rate and dwell time in State 1, while significantly higher fraction rate and dwell time in State 2. LBP Pre: patients before performing maneuver; LBP Post: patients after performing maneuver; HC: healthy control. LBP Sess1: patients in Session 1; LBP Sess2: patients in Session 2. N=90 and 74 for cLBP patients and HCs in Dataset 1, N=30 and 30 for cLBP patients and HCs in Dataset 2. Data are presented by mean ± standard error of mean (SEM). Asterisks represent two-sided $p_{FDR}<0.05$ for two-sample (between LBP and HC) and paired-sample (between LBP pre and post, between LBP sess1 and sess2) t tests. Each circle represents the value (fraction rate or dwell time) for an individual.

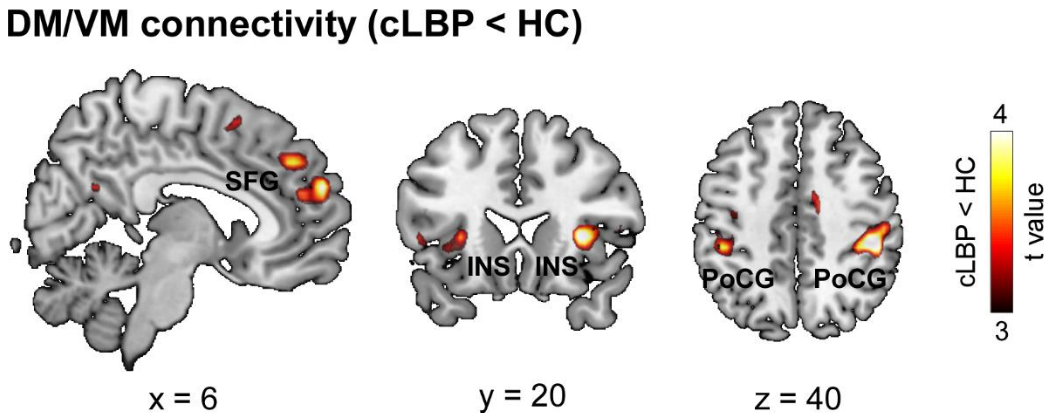

Supplementary Figure 11. Group differences of dorsal medial/ventral medial (DM/VM) nucleus-whole brain static connectivity. We found that cLBP patients had lower connectivity between DM/VM and superior frontal gyrus (SFG), insula (INS), and postcentral gyrus (PoCG).

Supplementary Note 3: Dynamic thalamocortical connectivity using FSL sub-thalamic atlas

We used the 7 FSL sub-thalamic nuclei, which subdivides the thalamus based on structural connectivity (estimated using probabilistic diffusion tractography on DTI data), into the following seven large cortical areas: primary motor, somatosensory, occipital, prefrontal, pre-motor, parietal, and temporal (https://fsl.fmrib.ox.ac.uk/fsl/fslwiki/Atlases) [3]. We performed the same analyses as detailed in the manuscript (seed-based whole-brain dFNC was estimated using seed-based correlation analysis between the time courses of these sub-thalamic regions and time courses of other voxels in the brain in State 1 and State 2; statistical comparisons were performed between cLBP and HCs and between pre-maneuver and post-maneuver).

Within State 2, in addition to the results we reported using the ICA-derived masks, we also found significantly decreased connectivity between the temporal thalamic nucleus and right middle temporal gyrus (MTG) after the maneuver.

Within State 1, in addition to the results we reported using the ICA-derived masks, we found that cLBP patients had significantly higher connectivity between the occipital thalamic nucleus and posterior cingulate cortex (PCC) and between the parietal thalamic nucleus and supramarginal gyrus (SMG), while decreased connectivity between the prefrontal thalamic nucleus and insula/inferior frontal gyrus (IFG). We also found that the maneuver could increase connectivity between the pre-motor thalamic nucleus and supplementary motor area (SMA).

In summary, our findings using the ICA parcellated thalamus were consistent with findings using the FSL thalamic atlas. Specifically, the results from the primary motor thalamic nucleus (the principal output of VL is primary motor cortex) and somatosensory thalamic nucleus (the principal output of VPL is PoCG and ParaCL) were consistent with the results obtained from VL/VPL. Results from prefrontal thalamic nucleus (the principal output of DM is prefrontal cortex) were consistent with the results obtained from

DM/VM. Nevertheless, there were some other thalamocortical connections that may have accounted for the connectivity difference between the two groups.

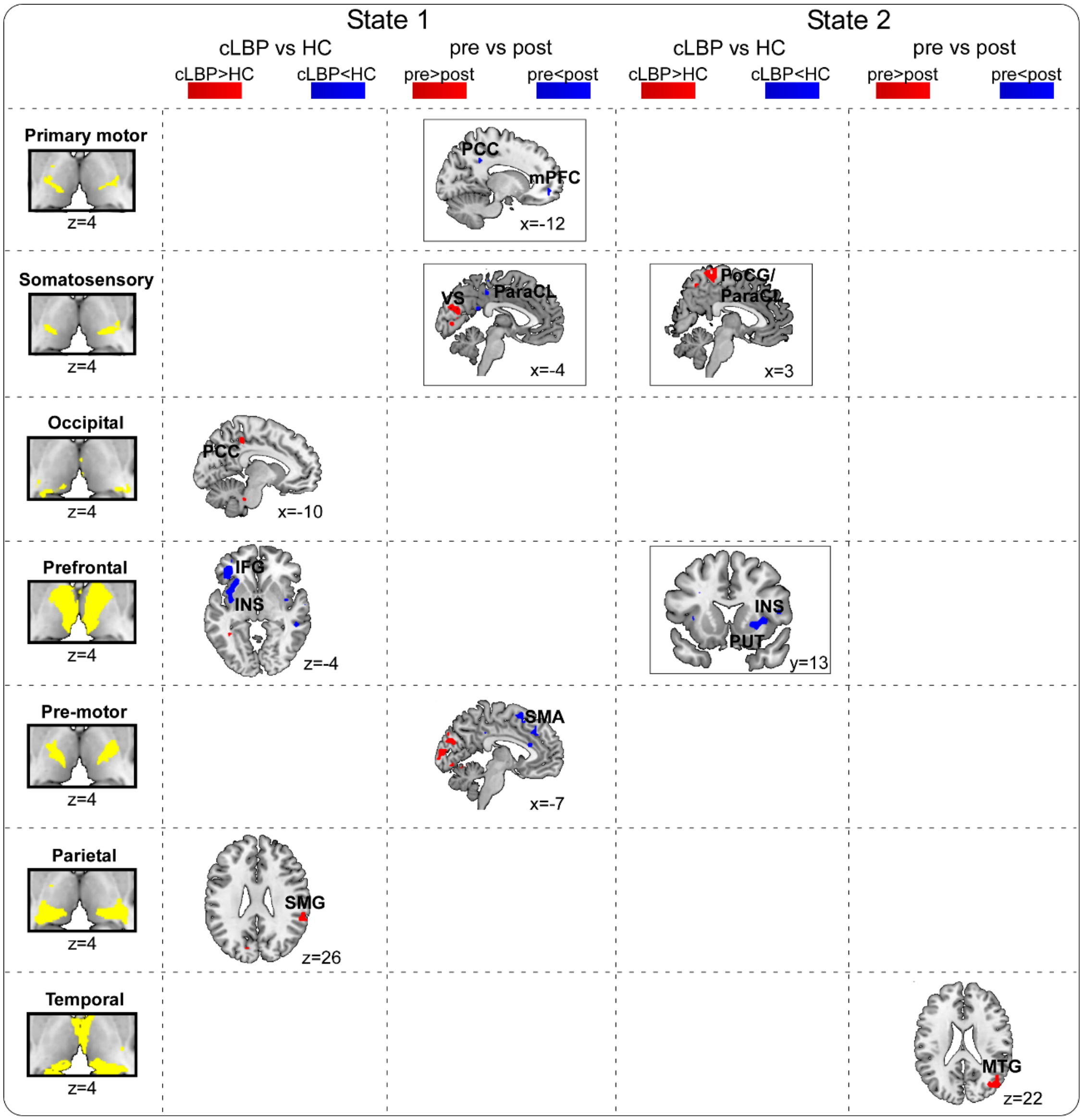

Supplementary Figure 12. Seed-based analysis using 7 FSL sub-thalamic atlas. Solid squares indicate findings consistent with the two ICA-derived masks. We performed two sample t-test when comparing cLBP and HCs, and paired-sample t-test when comparing pre- and post-maneuver. Two-sided p values were reported. Statistical maps were thresholded at $p < 0.005$ at voxel level and $p_{\mathrm{FDR}} < 0.05$ at cluster level.

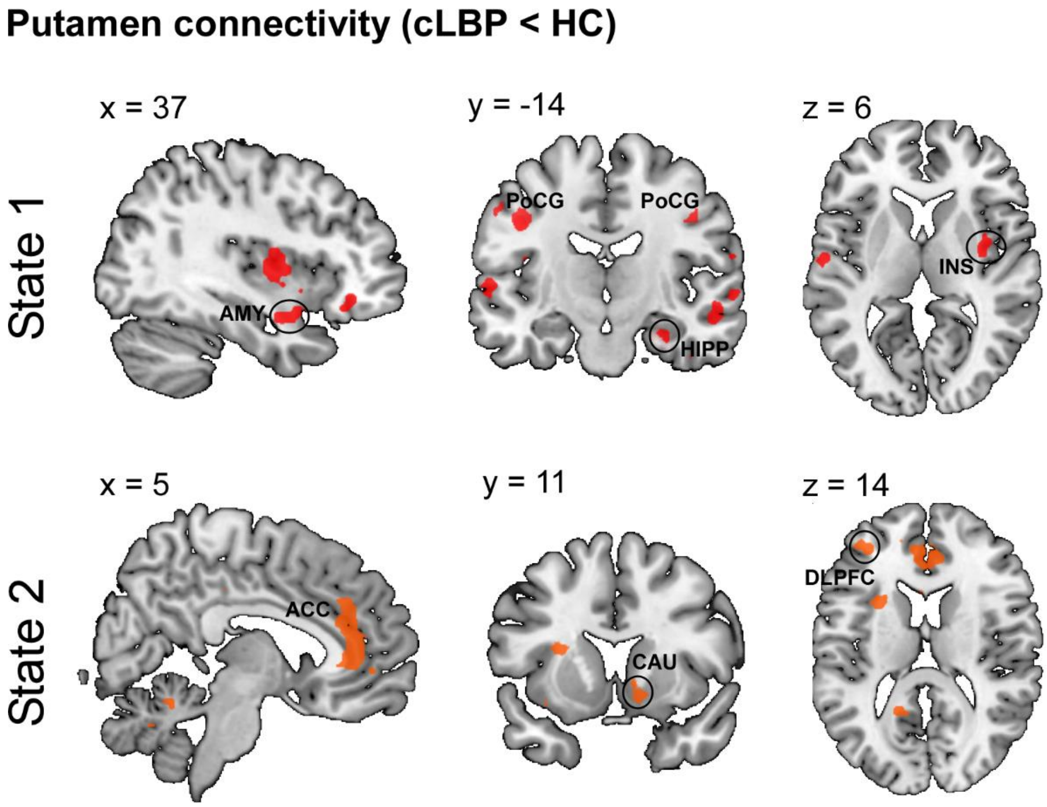

Supplementary Figure 13. Group differences of putamen-whole brain dFNC in two dynamic states. In State 1, cLBP patients had lower connectivity between the putamen and amygdala (AMY), postcentral gyrus (PoCG), hippocampus (HIPP), and insula (INS). In State 2, patients had lower connectivity between the putamen and anterior cingulate cortex (ACC), caudate (CAU), and dorsalateral prefrontal cortex (DLPFC).

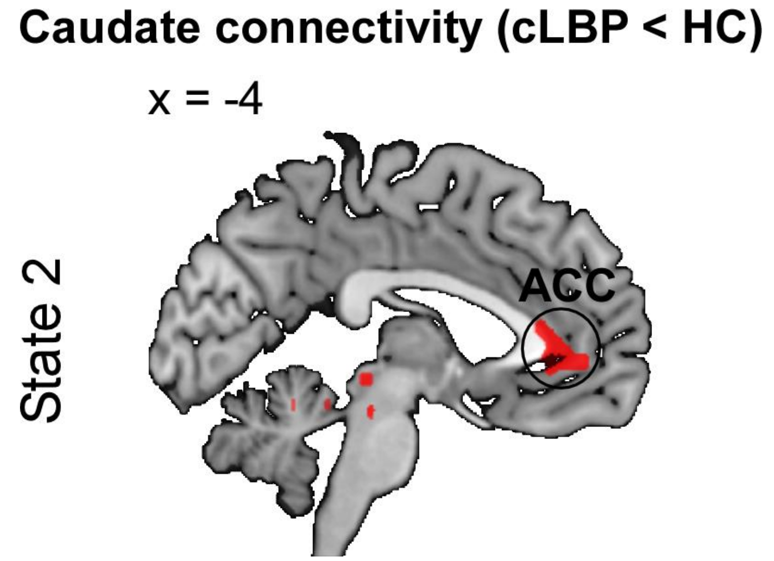

Supplementary Figure 14. Group differences of caudate-whole brain dFNC in two dynamic states. In State 2, cLBP patients had lower connectivity between the caudate and anterior cingulate cortex (ACC).

Supplementary Note 4: Vigilance-related analyses

In Falahpour et al., authors developed an fMRI-based spatial template to evaluate instantaneous level of vigilance during the scan [4]. We used this template (i.e., whole-brain spatial patterns for 'non-caffeine eyes-open' condition) to assess the vigilance level of our participants. Technical details can be found within the paper [4]. In brief, we calculated the spatial correlation between the template and fMRI BOLD at each time point, resulting in a time course of coefficients reflecting the putative vigilance index. The standard deviation of the vigilance time series was used to summarize each individual's fluctuations in vigilance level during the resting-state fMRI scan. In addition, since the vigilance template-based approach does not give information about a subject's mean vigilance level during a scan, we also used global signal amplitude (GSA), which has demonstrated to be strongly related to mean vigilance level [5,6]. We calculated these two measures for two different states (i.e., calculated standard deviation of vigilance time series and GSA within Gaussian windows correspond to State 1 and State 2, respectively). We used two-way ANOVA with factors of group (i.e., pre-maneuver cLBP, post-maneuver cLBP, and HCs) and state (i.e., State 1 and State 2) to assess vigilance levels. We did not find any significant main effects of group ($F=1.58$, $p=0.21$), state ($F=2.19$, $p=0.14$), and their interaction ($F=1.73$, $p=0.18$) on the mean level of vigilance (i.e., GSA). We also did not find any significant main effects of group ($F=0.86$, $p=0.42$), state ($F=0.89$, $p=0.35$), and their interaction ($F=0.16$, $p=0.85$) on fluctuations of vigilance.

In order to further evaluate vigilance level in the dataset, we performed an additional control analysis by using the proposed automatic sleep staging support vector machine (SVM) classifier described in Tagliazucchi E, et al. [7,8] to examine if there were drowsiness/vigilance differences between cLBP patients and HCs, as well as between State 1 and State 2. In other words, we argued that 1) if the SVM classifier could significantly classify cLBP patients and HCs, then these two cohorts of participants might have had systematic differences in drowsiness during the scans; 2) if the SVM classifier could significantly classify State 1 and State 2, then these two states of functional connectivity may be driven by the differences in drowsiness during the scans. We followed the procedure in Tagliazucchi et al. by parcellating brain

activity into 22 ROIs, and extracted rsFC features. Details of coordinates of ROIs and patterns can be found in Table 3 and Figure S2 within the manuscript [8]. For the discrimination of average drowsiness/vigilance level between the two cohorts, rsFC features were calculated using the entire scans between ROIs. For the discrimination of drowsiness/vigilance level between two states, the dFNC features were calculated within Gaussian windows corresponding to State 1 and State 2 respectively. The SVM classifier was used based on the leave-one-out cross-validation strategy (LOOCV). Nonparametric permutation tests (1000 times) were employed to estimate the statistical significance of the observed classification accuracy. In permutation testing, we randomly permuted the class labels of the data prior to training. LOOCV was then performed on the permuted class dataset and the procedure was repeated 1000 times. If the classifier trained on real class labels had an accuracy exceeding the 95% confidence interval generated from the accuracies of the classifiers trained on randomly relabeled class labels, this classifier was considered to be well-performing.

As shown in Supplementary Figure 15, the SVM classifier could not significantly discriminate cLBP patients from HCs (Accuracy = 53.2%; $p = 0.30$, permutation testing), nor could it discriminate State 1 from State 2 (Accuracy = 55.4%; $p = 0.10$, permutation testing). Therefore, we believe that there was not a systematic difference in drowsiness/vigilance levels between cLBP and HCs or between State 1 and State 2.

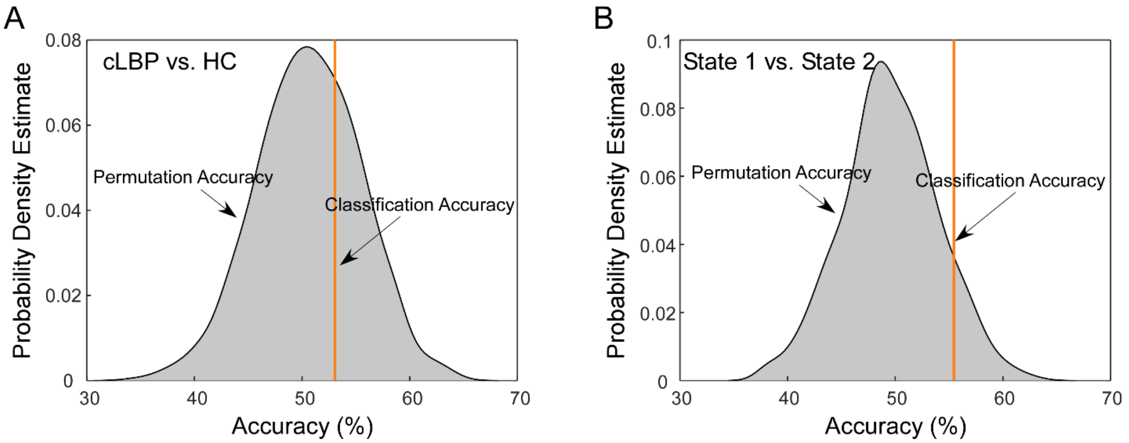

Supplementary Figure 15. Classification accuracies for discriminating (A) cLBP and HCs, and (B) State 1 and State 2, using the automatic sleep staging SVM classifier.

Supplementary Note 5: Head-motion related analyses

We used the framewise displacement (FD) to express instantaneous head motion:

$$FD_i = |\Delta d_{ix}| + |\Delta d_{iy}| + |\Delta d_{iz}| + |\Delta \alpha_i| + |\Delta \beta_i| + |\Delta \gamma_i| \quad (1)$$

where $\Delta d_{ix} = d_{(i-1)x} - d_{ix}$ and similarly for other rigid body parameters $[d_{ix}\ d_{iy}\ d_{iz}\ \alpha_i\ \beta_i\ \gamma_i]$.

We used maximal FD as summarized head motion value for each participant. In Dataset 1, we did not observe any significant difference in head motion across cLBP patients before maneuver, after maneuver, and HCs (0.57 ± 0.04, 0.59 ± 0.04, and 0.51 ± 0.06 respectively [mean ± standard error of mean]; $p > 0.05$ for all comparisons using t-test; Figure S16). The head motion was not correlated with pain severity (r= 0.02, p =0.86), fraction rate (r =0.04, p =0.56), and dwell time (r =0.003, p =0.97). Similar results were also found in Dataset 2 showing that head motion did not differ between cLBP patients in Session 1 (0.74 ± 0.11), in Session 2 (0.87 ± 0.08), and HCs (0.88 ± 0.08), as well as in Dataset 3 showing that head motion did not differ between cLBP patients (0.59 ± 0.10) and HCs (0.56 ± 0.06) (Figure S16).

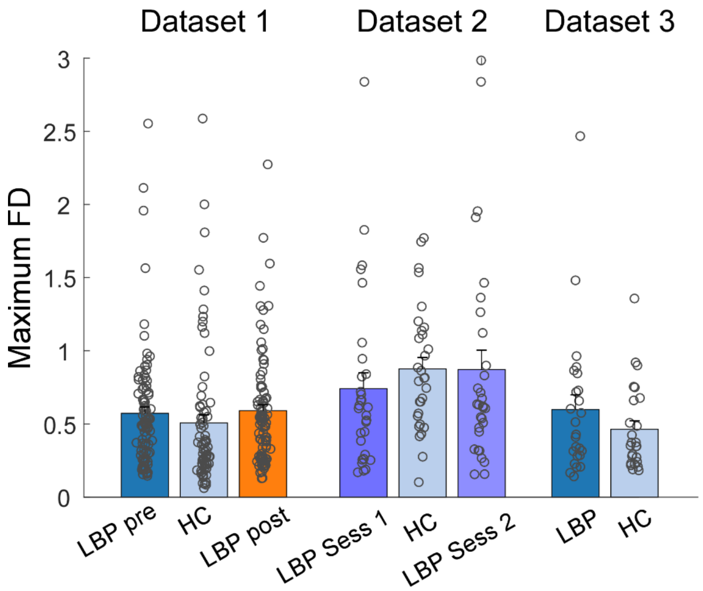

Supplementary Figure 16. Summarized head motion values (mean ± SEM) for different groups. Errorbars represent the standard error of the mean. Each circle represents the data (maximal FD) for an individual. N=90 and 74 for cLBP patients and HCs in Dataset 1, N=30 and 30 for cLBP patients and HCs in Dataset 2, N=25 and 25 for cLBP patients and HCs in Dataset 3).

Supplementary Note 6: Inclusion/Exclusion criteria

Inclusion Criteria:

a) Volunteers 18-60 years of age.

b) Meet the Classification Criteria of chronic LBP (having low back pain for more than 6 months).

c) At least 4/10 clinical pain on the 11-point LBP intensity scale.

d) Must have had a prior evaluation of their low back pain by a health care provider, which may have included radiographic studies. Documentation of this evaluation will be sought from Partners or outside medical records and kept in the subject's research record.

Exclusion Criteria:

a) Specific causes of back pain (e.g. cancer, fractures, spinal stenosis, infections).

b) Complicated back problems (e.g. back surgery, medicolegal issues).

c) Conditions making study participation difficult (e.g. paralysis, psychoses, or other severe psychological problems).

d) The intent to undergo surgery during the time of involvement in the study.

e) History of cardiac, respiratory, or nervous system disease that, in the investigator's judgment, precludes participation in the study because of a heightened potential for adverse outcomes. For example: asthma or claustrophobia.

f) Presence of any contraindications to MRI scanning. For example: cardiac pacemaker, metal implants, fear of closed spaces, pregnancy.

g) Use of prescription opioids greater than 60 mg morphine equivalents per day or steroids for pain.

h) Active substance abuse disorder in the past 24 months, as determined by self-report and/or urine toxicology.

Supplementary Methods: Dynamic functional network connectivity (dFNC) state analysis

The framework of characterizing dynamic rsFC to detect atypical functional dynamics in chronic pain is shown in Figure S17. In step 1, we conducted a group independent component analysis (GICA) with a reference template (identified from large sample datasets of Human Connectome Project and Genomics Superstruct Project) to decompose whole brain resting-state fMRI data into multiple independent components (Supplementary Figure 17.1). Following GICA, we selected intrinsic component networks (ICNs) from the independent components according to their spatial activation maps. In step 2, we calculated dFNC among ICNs using a sliding window approach with graphic LASSO (Supplementary Figure 17.2). In step 3, we conducted a k-means-based hard clustering on the dFNC estimates to identify distinct FC states (dFNC states) and the frequency of their occurrence (fraction rate and dwell time), which together allowed us to construct a profile of the brain connectivity dynamics during a given period for each participant (Supplementary Figure 17.3). The profile derived for one participant is shown in Supplementary Figure 17.3C. This profile captures changes in the dFNC state for each participant during a 6-min scan. In step 4, we applied graph theory measures on different dFNC states to demonstrate the efficiency of information transfer in dynamic brain networks.

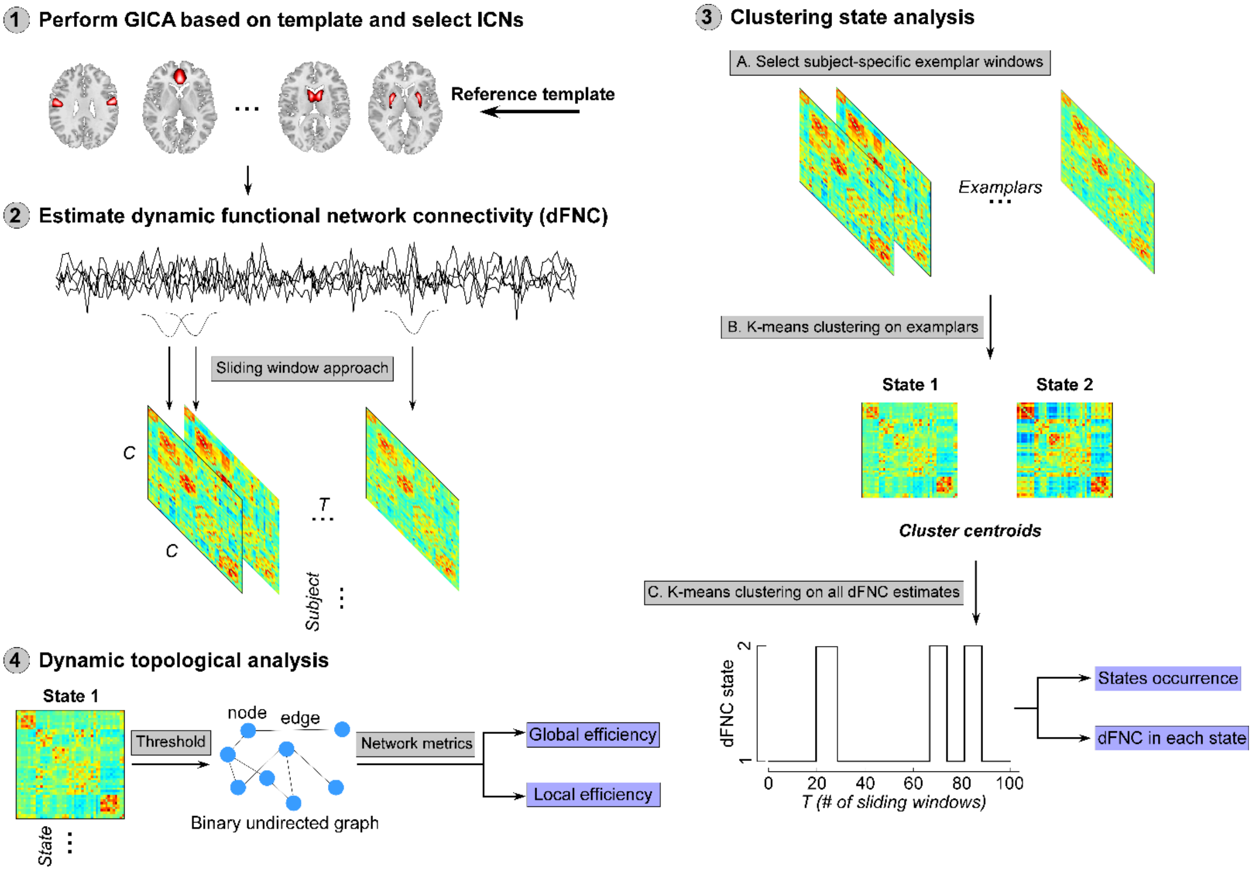

Supplementary Figure 17. Analysis framework to study dynamic functional network connectivity (dFNC) in patients with chronic pain. Four major steps were included: 1) perform group independent component analysis (GICA) with a spatial reference template and select intrinsic connectivity networks (ICNs); 2) estimate dFNC; 3) perform clustering state analysis; and 4) perform dynamic topologic analysis.

Group independent component analysis

The group independent component analysis (GICA) was performed using GIFT toolbox (mialab.mrn.org/software/gift/). We used the group template as a reference within a spatially constrained ICA algorithm to compute individual spatial maps and time-courses for all three datasets. In GICA, principle component analysis (PCA) was performed prior to ICA (this step was included in the GICA framework and implemented by GIFT toolbox). In PCA, the global mean signal per time point was removed as the standard PCA processing step during subject-level PCA reduction. This technical point is detailed and Allen et al [9]. Additional postprocessing steps on time courses were conducted to remove remaining noise sources, including 1) detrending linear, quadratic, and cubic trends; 2) conducting multiple

regressions of the six realignment parameters and their temporal derivatives; 3) despiking detected outliers; and 4) low-pass filtering with a cutoff frequency of 0.15 Hz.

dFNC estimation

For each subject, we estimated dFNC between the time courses (119 time points, 159 time points, and 158 time points for Dataset 1, Dataset 2, and Dataset 3) of ICNs using a sliding window approach. We used a tapered window, which was obtained by convolving a rectangle (window size = 20 × TRs = 60 s) with a Gaussian ($\sigma = 3$) to localize the dataset at each time point. The window was slid in steps of 1 TR, resulting in T = 99/139 total windows (Dataset 1/Dataset 2). The window size was selected based on the fMRI TR and according to previous studies showing that a window size in the range of 30 s to 1 min is a reasonable choice for capturing dynamic patterns in functional connectivity [10,11]. We calculated the covariance matrix with windowed data to measure the dFNC between ICNs. The dFNC estimates of all windows for each subject were concatenated to form a $C \times C \times T$ array (where C denotes the number of ICNs and T denotes the number of windows), which represented the changes in functional connectivity between ICNs as a function of time.

The Dataset 3 used for cross-site validation had a TR of 2.5 secs. Therefore, we used a window size of 24 TRs (i.e., 60 s). Other procedures were identical to those used in the first two datasets.

State cluster analysis

To assess the dFNC patterns that reoccur over time, we conducted hard clustering on the dFNC estimates. We used the K-means clustering method with L1 distance function to cluster the windowed dFNC estimates into a set of separate clusters. Similar to EEG microstate analysis [9], we first chose subject-specific exemplars, which are the time windows with local maxima in FNC variance across all connectivity pairs.

Then, we conducted the k-means clustering on these exemplars of all patients and controls and combined and repeated it 100 times (with random initialization of centroid position) to obtain the group cluster centroids (functional dFNC states). The optimal number of centroid states was estimated by the silhouette method, which measures how similar an object is to its own cluster compared to other clusters. The optimal number of clusters was determined as $k = 2$, with each cluster representing a functional dFNC state. We used the obtained group centroids as the initial centroids to cluster each subject's windowed dFNC.

Clustering the dFNC of the Dataset 3 was based on calculating the Euclidian distance between each dFNC and the centroids of the two states identified from Dataset 1.

Dynamic topological analysis

We applied graph theory analysis to investigate the topological organization of the functional dFNC states and compare it between cLBP and HCs. For this analysis, we defined those GICA-identified 45 ICNs as nodes and the dFNC between them as edges, and we constructed a 45×45 connectivity matrix for each subject and each state. The graph theory analysis was performed using the GRETNA software (http://www.nitrc.org/projects/gretna) [12]. Similar to previous studies [12,13], we first applied a sparsity threshold S (the ratio of the number of actual edges to the maximum possible number of edges in a network) to sparsify all connectivity matrices that ranged from 0.1 to 0.35 with a step of 0.05 based on the ranges of previous studies [14–16]. We then generated an undirected and unweighted adjacency matrix for each subject and each state by setting edges as 1 or 0 (edges were designated as 1 if an edge between node i and node j was larger than the threshold we selected, and 0 if it was smaller than the threshold; absolute values of connectivity were considered).

For the adjacency matrix at each sparsity threshold, we calculated global and local network efficiency to investigate local and global information transfer [17]. In brief, we defined efficiency as inversely proportional to the harmonic mean of the shortest distance (number of edges) between all possible pairs of nodes. The

global efficiency was the average efficiency across all node pairs, while the local efficiency was the average of the nodal local efficiency within neighbors of the node. To avoid the specific selection of a threshold, we applied an area under the curve (AUC) approach, which has been widely used in previous studies [14,15]. For each topological measure, we calculated the AUC within the sparsity range and compared the AUC between cLBP and HCs for each dynamic state.

Supplementary References

1. Du, Y. & Fan, Y. Group information guided ICA for fMRI data analysis. *Neuroimage* **69**, 157–197 (2013).
2. Yeo, T. *et al.* The organization of the human cerebral cortex estimated by intrinsic functional connectivity. *J. Neurophysiol.* **106**, 1125–65 (2011).
3. Behrens, T. E. J. *et al.* Non-invasive mapping of connections between human thalamus and cortex using diffusion imaging. *Nat. Neurosci.* **6**, 750–757 (2003).
4. Falahpour, M., Chang, C., Wong, C. W. & Liu, T. T. Template-based prediction of vigilance fluctuations in resting-state fMRI. *Neuroimage* **174**, 317–327 (2018).
5. Wong, C. W., DeYoung, P. N. & Liu, T. T. Differences in the resting-state fMRI global signal amplitude between the eyes open and eyes closed states are related to changes in EEG vigilance. *Neuroimage* **124**, 24–31 (2016).
6. Wong, C. W., Olafsson, V., Tal, O. & Liu, T. T. The amplitude of the resting-state fMRI global signal is related to EEG vigilance measures. *Neuroimage* **83**, 983–990 (2013).
7. Tagliazucchi, E. & Laufs, H. Decoding Wakefulness Levels from Typical fMRI Resting-State Data Reveals Reliable Drifts between Wakefulness and Sleep. *Neuron* **82**, 695–708 (2014).
8. Tagliazucchi, E. *et al.* Automatic sleep staging using fMRI functional connectivity data. *Neuroimage* **63**, 63–72 (2012).
9. Allen, E. A., Damaraju, E., Eichele, T., Wu, L. & Calhoun, V. D. EEG Signatures of Dynamic Functional Network Connectivity States. *Brain Topogr.* **31**, 101–116 (2018).
10. Hutchison, R. M. *et al.* Dynamic functional connectivity: Promise, issues, and interpretations. *Neuroimage* **80**, 360–378 (2013).
11. Damaraju, E. *et al.* Dynamic functional connectivity analysis reveals transient states of dysconnectivity in schizophrenia. *NeuroImage Clin.* **5**, 298–308 (2014).
12. Wang, J. *et al.* GRETNA: a graph theoretical network analysis toolbox for imaging connectomics. *Front. Hum. Neurosci.* **9**, 386 (2015).
13. Keown, C. L. *et al.* Network Organization Is Globally Atypical in Autism: A Graph Theory Study of Intrinsic Functional Connectivity. *Biol. Psychiatry Cogn. Neurosci. Neuroimaging* **2**, 66–75 (2017).
14. Kim, J. *et al.* Abnormal intrinsic brain functional network dynamics in Parkinson's disease. *Brain* **140**, 2955–2967 (2017).
15. Zhang, J. *et al.* Disrupted Brain Connectivity Networks in Drug-Naive, First-Episode Major Depressive Disorder. *Biol. Psychiatry* **70**, 334–342 (2011).
16. Hashmi, J. A. *et al.* Dexmedetomidine Disrupts the Local and Global Efficiencies of Large-scale Brain Networks. *Anesthesiology* **126**, 419–430 (2017).
17. Rubinov, M. & Sporns, O. Complex network measures of brain connectivity: Uses and interpretations. *Neuroimage* **52**, 1059–1069 (2010).
