# Supplementary material for: Distinct thalamocortical network dynamics are associated with the pathophysiology of chronic low back pain
Source: Nat Commun. 2020 Aug 7;11:3948. doi: 10.1038/s41467-020-17788-z (PMC7414843; doi:10.1038/s41467-020-17788-z)
Supplement: Supplementary file 2 — Reporting Summary [file 41467_2020_17788_MOESM2_ESM.pdf]

nature research

Corresponding author(s): Jian Kong

Last updated by author(s): Jul 16, 2020

Reporting Summary

Nature Research wishes to improve the reproducibility of the work that we publish. This form provides structure for consistency and transparency in reporting. For further information on Nature Research policies, see our Editorial Policies and the Editorial Policy Checklist.

Statistics

For all statistical analyses, confirm that the following items are present in the figure legend, table legend, main text, or Methods section.

n/a	Confirmed	
☐	☒	The exact sample size (*n*) for each experimental group/condition, given as a discrete number and unit of measurement
☐	☒	A statement on whether measurements were taken from distinct samples or whether the same sample was measured repeatedly
☐	☒	The statistical test(s) used AND whether they are one- or two-sided<br>*Only common tests should be described solely by name; describe more complex techniques in the Methods section.*
☐	☒	A description of all covariates tested
☐	☒	A description of any assumptions or corrections, such as tests of normality and adjustment for multiple comparisons
☐	☒	A full description of the statistical parameters including central tendency (e.g. means) or other basic estimates (e.g. regression coefficient) AND variation (e.g. standard deviation) or associated estimates of uncertainty (e.g. confidence intervals)
☐	☒	For null hypothesis testing, the test statistic (e.g. *F*, *t*, *r*) with confidence intervals, effect sizes, degrees of freedom and *P* value noted<br>*Give P values as exact values whenever suitable.*
☒	☐	For Bayesian analysis, information on the choice of priors and Markov chain Monte Carlo settings
☒	☐	For hierarchical and complex designs, identification of the appropriate level for tests and full reporting of outcomes
☐	☒	Estimates of effect sizes (e.g. Cohen's *d*, Pearson's *r*), indicating how they were calculated

Our web collection on statistics for biologists contains articles on many of the points above.

Software and code

Policy information about availability of computer code

Data collection	Siemens Skyra Syngo, GE Signa HDxt, eprime2.0
Data analysis	MATLAB R2018b, CONN toolbox v17f for fMRI preprocessing, ART (incorporated in CONN) for detecting head motion, GIFT toolbox v3.0b for ICA, GRETNA toolbox v2.0 for calculating graphical measures, and custom matlab codes are available in github (https://github.com/yihengtu/Dynamic-connectivity-for-fMRI.git).

For manuscripts utilizing custom algorithms or software that are central to the research but not yet described in published literature, software must be made available to editors and reviewers. We strongly encourage code deposition in a community repository (e.g. GitHub). See the Nature Research guidelines for submitting code & software for further information.

Data

Policy information about availability of data

All manuscripts must include a data availability statement. This statement should provide the following information, where applicable:

- Accession codes, unique identifiers, or web links for publicly available datasets
- A list of figures that have associated raw data
- A description of any restrictions on data availability

The MRI data are from multiple sources. Not all participants gave their permission to share their data with the public. Some datasets are part of longitudinal studies that will generate more than one manuscript. The data will eventually be made available, with the permission of the participants, once these manuscripts are completed. Reasonable requests can be sent to the corresponding author (J.K.). The data used for deriving the group template of ICNs are available from HCP website (https://www.humanconnectome.org/) and GSP website (https://dataverse.harvard.edu/dataverse/GSP). The group template of ICNs derived from HCP and GSP for spatially constrained ICA can be requested from V.C. (vcalhoun@gsu.edu). Source data are provided with this paper.

Field-specific reporting

Please select the one below that is the best fit for your research. If you are not sure, read the appropriate sections before making your selection.

☒ Life sciences ☐ Behavioural & social sciences ☐ Ecological, evolutionary & environmental sciences

For a reference copy of the document with all sections, see nature.com/documents/nr-reporting-summary-flat.pdf

Life sciences study design

All studies must disclose on these points even when the disclosure is negative.

Sample size

The study included three independent datasets with fMRI data from a total of 145 chronic low back pain patients and 129 matched healthy controls. In addition, 120 chronic low back pain patients had two separated fMRI scans (either before and after pain exacerbation, or test-retest in two weeks). Compared to recent studies investigating the functional abnormalities in chronic pain patients, for example, in Lopez-Sola et al: 37 fibromyalgia patients vs 37 healthy controls; in Tu et al: 89 migrainuers vs 70 healthy controls; in Kutch et al: 110 chronic pelvic pain patients vs 107 healthy controls; in Zhang et al., 90 chronic low back pain patients vs 74 healthy controls; in Cottam et al., 25 chronic osteoarthritis pain patients vs 19 healthy controls, the sample size was considered as sufficiently large to detect group differences between patients and healthy controls.

References:

Lopez-Sola et al., Towards a neurophysiological signature for fibromyalgia, Pain 158, 34-47 (2017)

Tu et al., Abnormal thalamocortical network dynamics in migraine, Neurology 92, e2706-e2716 (2019)

Kutch et al., Brain signature and functional impact of centralized pain: a Multidisciplinary Approach to the Study of Chronic Pelvic Pain (MAPP) Network Study, Pain 158, 1979-1991 (2017).

Zhang et al., Identifying brain regions associated with the neuropathology of chronic low back pain: a resting-state amplitude of low-frequency fluctuation study, British J Anaesthesia 123, e303-e311 (2019)

Cottam et al., Altered connectivity of the right anterior insula drives the pain connectome changes in chronic knee osteoarthritis, Pain 159, 929-938 (2018).

Data exclusions

The inclusion and exclusion criteria were pre-established. In dataset 1, 14 of 90 chronic low back pain patients had the same or decreased levels of pain intensity after pain exacerbation maneuver. These 14 patients were excluded from the following analyses comparing dynamic resting-state functional connectivity and thalamocortical networks between low pain and exacerbated pain conditions.

Replication

We included two independent datasets (dataset 2 and dataset 3) for validation and quality control. In dataset 2, we included 30 chronic low back pain patients and 30 healthy controls. These participants had two resting-state fMRI scans (separated by about 2 weeks) using the same MRI scanner as we used in dataset 1. In dataset 3, we included 25 chronic low back pain patients and 25 healthy controls from an independent site that performed a multi-source interference task before and after resting-state fMRI scans to control the effect of vigilance. The reoccurring dynamic connectivity states and the temporal properties (i.e., fraction rate and dwell time) of two states were replicated in both dataset 2 and dataset 3.

Randomization

Participants consisted of chronic low back pain patients and healthy controls. Since the study aimed to compare the brain differences between patients and controls, no randomization was required in the study. In all three datasets, all patients and healthy controls were matched in gender and age, therefore these two major covariates (when studying brain differences between two populations) were controlled.

Blinding

The study design required no blinding. Since we aimed to compare the brain differences between patients and controls, and the study did not include any interventions (e.g., treatment), the group information (patients or healthy controls) was not blinded during data collection and data analysis.

Reporting for specific materials, systems and methods

We require information from authors about some types of materials, experimental systems and methods used in many studies. Here, indicate whether each material, system or method listed is relevant to your study. If you are not sure if a list item applies to your research, read the appropriate section before selecting a response.

Materials & experimental systems

n/a	Involved in the study
☒	☐ Antibodies
☒	☐ Eukaryotic cell lines
☒	☐ Palaeontology and archaeology
☒	☐ Animals and other organisms
☐	☒ Human research participants
☒	☐ Clinical data
☒	☐ Dual use research of concern

Methods

n/a	Involved in the study
☒	☐ ChIP-seq
☒	☐ Flow cytometry
☐	☒ MRI-based neuroimaging

Human research participants

Policy information about studies involving human research participants

Population characteristics	The first cohort (Dataset 1) consisted of 90 chronic low back pain patients (age 34.5±9.0; 38 males) and 74 healthy controls (age 32.4±8.4; 31 males). The second cohort (Dataset 2) consisted of 30 chronic low back pain patients (age 37.2±11.0; 13 males) and 30 healthy controls (age 33.5±7.2; 17 males). The third cohort (Dataset 3) consisted of 25 chronic low back pain patients (age 48.0 ± 9.6; 7 males) and 25 healthy controls (age 44.3 ± 12.2; 9 males)
Recruitment	Dataset 1 and 2 were recruited by advertisements at MGH, BWH and Boston area.<br>Dataset 3 was recruited at the outpatient clinic of the affiliated hospital of Xian Jiao Tong University.<br>All patients in the three datasets met the similar inclusion criteria.<br>All participants (including low back pain patients and healthy controls) were recruited through flyers and email lists. Although we cannot eliminate self-selection bias completely, the self-selection should be similar between the patients and controls, thus it is unlikely for the potential bias to impact our results.
Ethics oversight	The Institutional Review Board (IRB) of Massachusetts General Hospital (MGH) approved the first two datasets, and the Research Ethics Committee of the Xian Jiao Tong University approved the third dataset. All experiments were performed in accordance with the guidelines set forth by the IRB for ethics and protection of human participants. All participants signed consent form before the start of the experiment.

Note that full information on the approval of the study protocol must also be provided in the manuscript.

Magnetic resonance imaging

Experimental design

Design type	Resting-state
Design specifications	In the first dataset, all cLBP patients underwent two resting-state fMRI scan sessions. After the first MRI session, patients stepped out of the scanner and performed pain-exacerbating maneuvers to increase their LBP so that we could investigate the brain activity/connectivity changes following temporary back pain intensification. After the maneuvers, which took about 10 minutes, patients entered the scanner for another identical MRI session. Healthy controls did not perform maneuvers and underwent only one MRI session.<br>In the second dataset, cLBP patients did not perform maneuvers, and they underwent two MRI sessions separated by about 2 weeks. No treatment was administered between the two scan sessions. Healthy controls underwent only one MRI session.<br>In the third dataset, cLBP patients and HCs performed a multi-source interference task (MSIT) before and after the MRI scan to increase their attention level. Two different types of trials appeared alternatively (i.e., Control-Interference-Control-Interference) with a total of 96 trials (48 trials for each task; completed in two blocks outside the MRI room) before resting-state MRI and a total of 192 trials (96 trials for each task; completed in four blocks inside the MRI room) after resting-state MRI. Stimulus and inter-stimulus intervals were 1.75 and 0 s, respectively.
Behavioral performance measures	No behavioral performance data was recorded during resting state fMRI scanning

Acquisition

Imaging type(s)	functional and structural
Field strength	3 tesla
Sequence & imaging parameters	In the first and second dataset, functional MRI data were acquired using a 32-channel radio frequency head coil with gradient echo planar imaging (repetition time: 3,000 ms, echo time: 30 ms, flip angle: 90°, slice thickness: 3 mm, interslice gap: 0.88 mm, FOV: 240 mm, and 44 slices). Participants in Dataset 1 and Dataset 2 had 6-minute and 8-minute resting-state fMRI scans, respectively. Structural MRI data were acquired using a multi-echo magnetization-prepared rapid gradient-echo sequence (repetition time: 2,500 ms, echo time: 1.69 ms, slice thickness: 1 mm, flip angle: 7°, FOV: 256 mm, and 176 slices).

In the third dataset, functional MRI data were acquired using a 8-channel radio frequency head coil with gradient echo planar imaging (repetition time: 2,500 ms, echo time: 30 ms, flip angle: 90°, slice thickness: 3 mm, interslice gap: 0 mm, FOV: 256 mm, and 50 slices). Participants had 6 min 35 sec resting-state fMRI scans. Structural MRI data were acquired using a fast spoiled gradient echo sequence (repetition time: 10.7 ms, echo time: 4.8 ms, slice thickness 1 mm, flip angle: 7°, FOV: 256 mm, and 140 slices).

Area of acquisition: Whole brain

Diffusion MRI: ☐ Used ☒ Not used

Preprocessing

Preprocessing software: fMRI data were preprocessed using CONN toolbox version 17f. Preprocessing steps included a standard pipeline (functional realignment&unwarp, functional slice-timing correction, structural segmentation&normalization, functional normalization, functional outlier detection, and functional smoothing with a 5 mm full-width at half-maximum Gaussian kernel). The details of default preprocessing pipeline can be found in https://web.conn-toolbox.org/fmri-methods/preprocessing-pipeline.

Normalization: In CONN toolbox, Functional and anatomical data are normalized into standard MNI space and segmented into grey matter, white matter, and CSF tissue classes using SPM12 unified segmentation and normalization procedure. This procedure iteratively performs tissue classification, estimating the posterior tissue probability maps (TPMs) from the intensity values of the reference functional/anatomical image, and registration, estimating the non-linear spatial transformation best approximating the posterior and prior TPMs, until convergence. Direct normalization applies this unified segmentation and normalization procedure separately to the functional data, using the mean BOLD signal as reference image, and to the structural data, using the raw T1-weighted volume as reference image. Both functional and anatomical data are resampled to a default 180x216x180mm bounding box, with 2mm isotropic voxels for functional data and 1mm for anatomical data, using 4th order spline interpolation.

Normalization template: MNI

Noise and artifact removal: The first five scans were removed for signal equilibrium and participants' adaptation to the scanner's noise. Group independent analysis (GICA) identified spatial components related to head motion, white matter, and cerebrospinal fluid were discarded. Additionally, head motion was summarized as frame-wise displacement (FD) time series, according to Power's method. We used the maximal FD as summarized head motion value for each participant and we did not find any significant difference of head motion between patients and healthy controls in three datasets. Details can be found in Figure S16.

Volume censoring: Artifact detection tools (ART) was applied to detect motion during the resting state fMRI scan. Time points in subjects' images were marked as outliers if the global signal exceeded three standard deviations from the mean or if scan-to-scan motion deviation exceeded 0.5 mm. Those outliers, in together with linear and polynomial trends of 6 head motion parameters, were included as nuisance regressors during the denoising procedure in the post-processing steps of GICA.

Statistical modeling & inference

Model type and settings: To investigate the group difference of occurrence rates and graphical measures of two dynamic states, we used univariate two-sample t test to compare patients and healthy controls; paired-sample t test to compare patients in two sessions before and after maneuver; paired-sample t test to compare patients in two test-retest sessions. P values were false-positive discovery rate (FDR) corrected.

To investigate the abnormal thalamocortical networks in cLBP patients, we performed thalamus to whole brain connectivity analyses using two ICA derived thalamic nuclei. The resulted connectivity maps at individual levels were compared between cLBP patients and healthy controls using mass-univariate two-sample t test, as well as between cLBP patients before and after maneuver using mass-univariate paired-sample t test.

Effect(s) tested: No task or stimulus conditions were tested

Specify type of analysis: ☐ Whole brain ☐ ROI-based ☒ Both

Anatomical location(s): The primary ROI-based analysis was based on the group template of ICNs derived from HCP and GSP for spatially constrained ICA. We also validated the ROI-based results using the Yeo 7-network functional parcellation. The seed for thalamocortical network analysis was also derived from the ICN template. We also validated the results using the FSL sub-thalamic atlas.

Statistic type for inference (See Eklund et al. 2016): Mass-univariate statistical comparisons were thresholded at p<0.005 at voxel level

Correction: FDR correction was performed with a threshold of p<0.05 at cluster level.

Models & analysis

n/a	Involved in the study
☐	☒ Functional and/or effective connectivity
☐	☒ Graph analysis
☒	☐ Multivariate modeling or predictive analysis

Functional and/or effective connectivity

Pearson correlation

Graph analysis

We defined those GICA-identified 45 ICNs as nodes and the dFNC between them as edges, and we constructed a 45×45 connectivity matrix for each subject and each state. we first applied a sparsity threshold S (the ratio of the number of actual edges to the maximum possible number of edges in a network) to sparsify all connectivity matrices that ranged from 0.1 to 0.35 with a step of 0.05 based on the ranges of previous studies. We then generated an undirected and unweighted adjacency matrix for each subject and each state by setting edges as 1 or 0 (edges were designated as 1 if an edge between node i and node j was larger than the threshold we selected, and 0 if it was smaller than the threshold; absolute values of connectivity were considered).

For the adjacency matrix at each sparsity threshold, we calculated global and local network efficiency to investigate local and global information transfer. To avoid the specific selection of a threshold, we applied an area under the curve (AUC) approach, which has been widely used in previous studies. For each topological measure, we calculated the AUC within the sparsity range and compared the AUC between cLBP and HCs for each dynamic state.
